# The trends of maternal mortality ratios and cause pattern in 34 Chinese provinces, 1990–2017

**DOI:** 10.1186/s12889-022-13770-0

**Published:** 2022-07-16

**Authors:** Chang-li Li, Meng Jiang, Ke-cheng Huang, Jian Li, Li-gang Xu

**Affiliations:** 1 Department of FSTC Clinic, The First Affiliated Hospital, Zhejiang University School of Medicine, Hangzhou, 310003 China; 2 Emergency and Trauma Center, The First Affiliated Hospital, Zhejiang University School of Medicine, 310003 Hangzhou, China; 3 Department of Gynecology and Obstetrics, Tongji Hospital, Tongji Medical College, Huazhong University of Science and Technology, Wuhan, 430030 China; 4 Tongji Hospital, Tongji Medical College, Huazhong University of Science and Technology, Wuhan, 430030 China

**Keywords:** Maternal mortality ratios, Cause pattern, Spatiotemporal trends, China

## Abstract

**Background:**

Understanding the trends and causes to the burden of maternal deaths is a key requirement to further reduce the maternal mortality ratio (MMR), and devise targeted intervention policy. We aimed to evaluate the spatiotemporal trends of MMRs and cause patterns across the 34 provinces of China during 1990–2017.

**Methods:**

Using data from the Global Burden of Disease Study 2017, we calculated the levels and trends of total maternal deaths and MMR due to ten different causes through Bayesian multivariable regression model for pregnancies aged 10–54 years, and assessed the age and regional distribution over time.

**Results:**

China has experienced fast decline in MMR, dropped from 95.2 (87.8–102.3) in 1990 to 13.6 (12.5–15.0) in 2017, with an annualised rate of decline of 7.0%. In 1990, the range of MMRs in mainland China was 31.1 in Shanghai, to 323.4 in Tibet. Almost all provinces showed remarkable decline in the last two decades. However, spatial heterogeneity in levels and trends still existed. The annualised rate of decline across provinces from 1990 to 2017 ranged from 0.54% to 10.14%. Decline accelerated between 2005 and 2017 compared with between 1990 and 2005. In 2017, the lowest MMR was 4.2 in Zhejiang; the highest was still in Tibet, but had fallen to 82.7, dropped by 74.4%. MMR was highest in the 40–49 years age group in both 1990 and 2017. In 2017, haemorrhage and hypertensive disorders were the leading two specific causes for maternal deaths.

**Conclusions:**

MMRs have declined rapidly and universally across the provinces of China. Setting of associated interventions in the future will need careful consideration of provinces that still have MMR significantly higher than the national mean level.

**Supplementary Information:**

The online version contains supplementary material available at 10.1186/s12889-022-13770-0.

## Introduction

Reducing maternal mortality is challenging and has long been a global health priority. It is one of the eight targets in the United Nations (UN) Millennium Development Goals (MDG) framework [1], and a key goal of the Global Strategy for Women’s and Children’s Health launched by the UN Secretary-General in September, 2010 [2]. Over the past two decades, China has seen rapid progress in improving maternal health, pushing down the maternal mortality ratio (MMR; number of maternal deaths per 100 000 livebirths) at an annualised rate of 7.5% per year from 1990 to 2015, one of the fastest decreases in the world [3]. The national MMR fell from 114.2 in 1990 to 85.2 per 100 000 live births in 2000, and to 17.7 in 2015 [3].

The levels and trends of maternal mortality in China has recently been systematically reported using data from the national Annual Report System on Maternal and Child Health (ARMCH) during 1996–2015 [4]. This survey found substantial heterogeneity in MMR at the country level, the annualised rate of decline in maternal deaths across counties of China from 1996 to 2015 ranged from 4.4% to 12.9%, and 2838 of the 2852 (99.5%) counties had achieved the MDG 5 pace of decline. However, it did not document the cause pattern of maternal deaths and its differences in age groups. Understanding the causes to the burden of maternal deaths is a key requirement to further reduce the MMR, and devise targeted intervention policy. More recently, the Global Burden of Diseases, Injuries, and Risk Factors Study 2017 (GBD 2017) provided estimates of maternal causes of death as part of the analysis of all causes of death. In this study, we reported the spatiotemporal trends of maternal mortality by province and age groups, and tracked the key causes contributing to maternal deaths in China during 1990–2017, using data from GBD 2017.

## Materials and methods

### Data source

Data on the absolute number of maternal deaths, MMR and cause categories by the China province-level during 1990–2017 were obtained from the Maternal Health Atlas of GBD 2017 (publicly available online: https://maternalhealthatlas.org/). GBD defines maternal deaths as “any death of a woman (range from 10 to 54 years age) while pregnant or within one year of termination of pregnancy, irrespective of the duration and site of the pregnancy, from any cause related to or aggravated by the pregnancy or its management but not from accidental or incidental causes” [4–6]. According to the definition of GBD 2017, maternal deaths were disaggregated into to ten causes: maternal haemorrhage, maternal hypertensive disorders, ectopic pregnancy, indirect maternal deaths, late maternal deaths, maternal abortion and miscarriage, maternal obstructed labor and uterine rupture, maternal sepsis and other maternal infections, maternal deaths aggravated by HIV/AIDS, and other maternal disorders.

We present findings for 34 province­level administrative units of China: 23 provinces, 4 municipalities, 5 autonomous regions, and 2 special administrative regions (SARs), but they are all termed as “province” throughout the current article. We used standard GBD data processing procedures and analytical models to generate maternal mortality estimates. Detailed descriptions on these approaches can be found in previous GBD publications [4–6]. In this research, the discussion is mainly focused on the province­level burden of maternal mortality for mainland China.

### Statistical analyses

The MMR was estimated for the 34 provinces, by age and cause, from 1990 to 2017 using DisMod-MR 2.1, a Bayesian meta-regression tool developed for the GBD [3]. The final results consisted of cause fraction and number of maternal deaths due to each cause, by province, age group, and year. The MMRs for each 5-year age group were calculated between 15–49 years separately using age-specific livebirths. Because no standard estimates of birth rates were available for the age group 10–14 and 50–54 years, we just estimated the number of maternal deaths in the two age groups but did not calculate the MMR for them. The annualised rate of change (ARC) was calculated using the continuous rate-of-change formula [7] in each province separately for 1990–2005 and 2005–2017. ARC examination highlights overall trends, and allows identification of those provinces that have achieved MDG 5 (equivalent to a sustained 5.5% decrease per year).

The expected number of maternal deaths were probabilistically predicted based on the Socio-demographic Index (SDI) of each province during 1990–2017 [8]. The observed/expected maternal deaths (O/E) was calculated to explore whether the province doing better or worse than expected. The SDI is a combined measure of total fertility rates among women younger than 25 years, average educational attainment in the population older than 15 years, and lag­distributed income per person [8]. Each index is scaled from 0 (highest fertility, fewest years of schooling, and lowest income) to 1 (lowest fertility, most years of schooling, and highest income), and the geometric mean value of the three indexes produces a final index score of 0–1. SDI values reflect the degree of social development. The national SDI for China in 2017 was 0.71, ranged from 0.47 to 0.86 at the province level [9]. The methods used to calculate the SDI are described in detail in previous report [10].

GBD 2017 generated the 95% uncertainty intervals (UIs) for the cause-specific maternal deaths, MMR and annualised rates of change for China as a country. The values were generated from the mean of 1000 draws, and the 95% UIs were determined by the 2.5th and 97.5th centiles of the ordered draws. This study is compliant with the Guidelines for Accurate and Transparent Health Estimates Reporting (GATHER) [11].

## Results

### National and province-specific maternal mortality

**Fig. 1:**
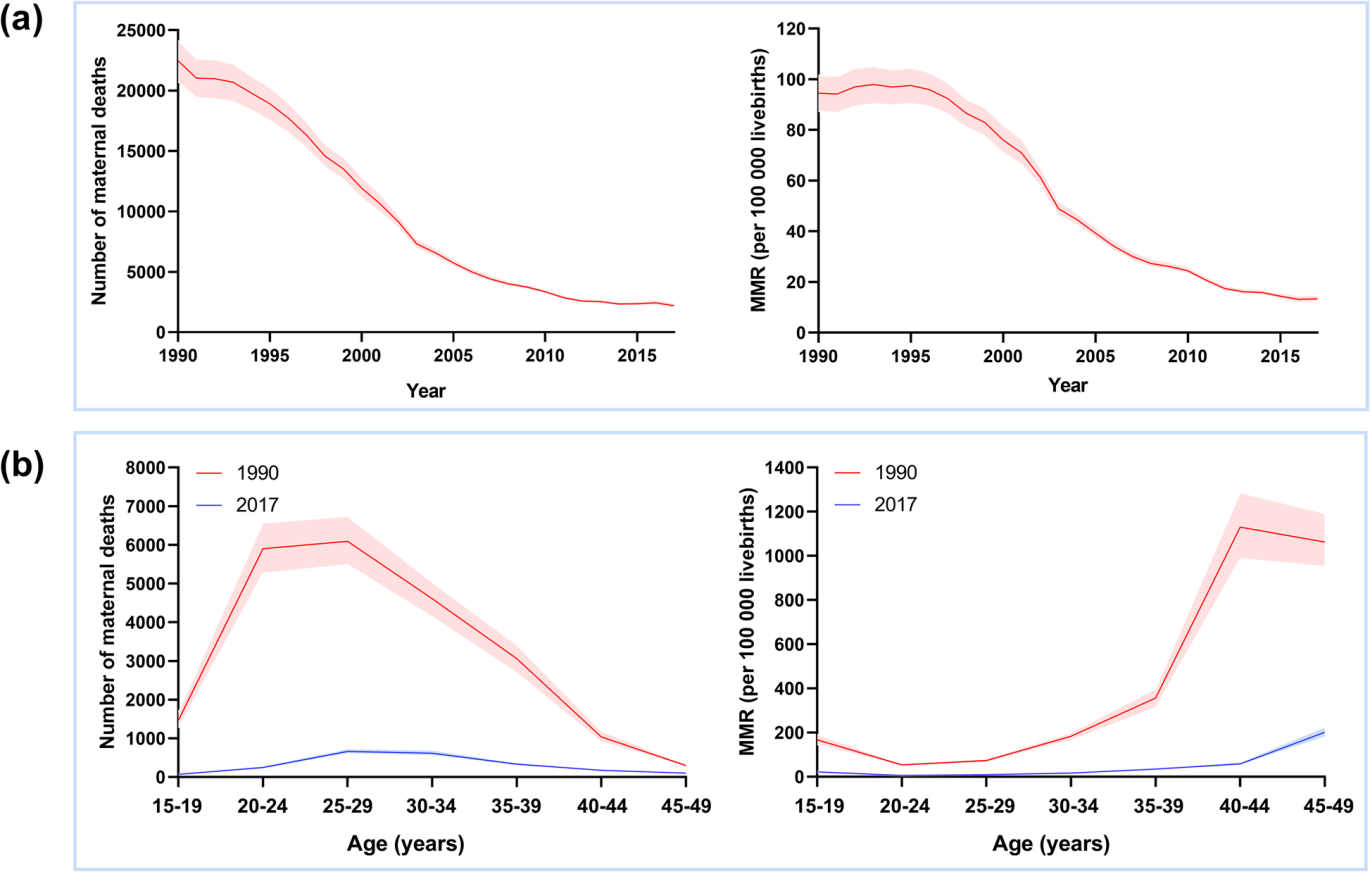
National results with 95% uncertainty interval for the **a** number of maternal deaths (left) and maternal mortality ratio (MMR; number of deaths per 100,000 livebirths; right) by year (1990–2017), and **b** national number of maternal deaths (left) and MMR (right) in 1990 and 2017, by age groups. Shaded areas show 95% uncertainty intervals

From 1990 to 2017, the total annual number of maternal deaths declined remarkably by 90.1% in China, from 22,615.2 (95% UI 20,867.8–24,304.2) to 2240.5 (95% UI 2058.0–2477.9); the MMR declined from 95.2 (95% UI 87.8–102.3) in 1990 to 13.6 (95% UI 12.5–15.0) in 2017, dropped by 85.7% (Table 1, Fig. 1a). The women aged 20–29 years had the greatest number of maternal deaths; however, MMR was lowest in these women but highest in the 40–49 years age groups in both 1990 and 2017 (Fig. 1b). Both the total number of deaths and MMR decreased significantly between 1990 and 2017 for all age groups (Fig. 1b). We used data of 2017 (Table 2) for estimating the proportions of maternal death in different age groups, and observed that 3.1% of maternal deaths happened in the group aged 15–19 years, 40.5% in women aged 20–29 years, and 56.0% in those aged 30 years and older, with the remainder 0.3% occurring in the group aged 10–14 years. The MMR in mothers aged 15–19 years in 2017 was 3.6 times higher than that in women aged 20–24 years, and 2.3 times higher than those aged 25–29 years. Notably, the MMR was 33.0 times higher for a woman aged 45–49 years (201.5, 95% UI 179.3–225.8) than for a woman aged 20–24 years (6.1, 95% UI 5.3–7.0).

**Table 1:** Provincial number of maternal deaths, MMR, annualized rates of change in MMR and percent MMR reduction between 1990 and 2017, and ratio of observed to expected maternal deaths

Province	Number of maternal deaths			MMR			Annualized rate of change in MMR 1990–2017(%)	MMR reduction between 1990 and 2017 (%)	Observed/expected maternal deaths based on SDI		
	**1990**	**2005**	**2017**	**1990**	**2005**	**2017**			**1990**	**2005**	**2017**
China overall	22,615.2	5783.4	2240.5	95.2	39.7	13.6	-7.0	85.7	0.27	0.25	0.29
Anhui	1366.9	244.3	85.0	92.2	28.7	10.0	-8.21	89.2	0.19	0.10	0.11
Beijing	112.8	41.9	22.8	66.8	25.5	8.9	-7.46	86.7	0.83	0.70	0.61
Chongqing	319.6	136.8	30.0	83.9	32.9	8.8	-8.37	89.5	0.19	0.15	0.14
Fujian	443.6	109.8	38.8	58.3	18.0	6.5	-8.13	88.9	0.16	0.13	0.17
Gansu	884.7	260.8	89.9	138.5	69.0	23.0	-6.65	83.4	0.28	0.21	0.20
Guangdong	897.8	396.1	164.3	61.6	24.1	9.6	-6.88	84.4	0.24	0.32	0.41
Guangxi	1314.3	210.9	91.5	178.7	51.0	14.7	-9.25	91.8	0.38	0.23	0.26
Guizhou	1765.1	325.1	102.3	304.2	137.9	30.9	-8.47	89.8	0.35	0.17	0.14
Hainan	136.4	41.4	20.9	82.4	30.9	14.2	-6.51	82.8	0.29	0.24	0.30
Hebei	620.0	285.7	131.4	46.2	29.2	12.2	-4.94	73.6	0.12	0.21	0.30
Heilongjiang	323.5	68.7	26.3	57.5	29.7	12.3	-5.7	78.6	0.17	0.12	0.12
Henan	1377.3	469.7	202.7	79.5	57.8	16.6	-5.81	79.1	0.17	0.23	0.28
Hong Kong	9.4	9.9	6.3	13.8	16.4	10.7	-0.93	22.5	0.24	0.42	0.48
Hubei	564.7	111.5	52.6	55.8	29.6	10.2	-6.28	81.7	0.14	0.10	0.14
Hunan	1287.3	228.9	102.7	131.2	52.0	16.6	-7.65	87.3	0.26	0.18	0.22
Inner Mongolia	432.7	86.2	25.3	87.3	27.6	9.2	-8.32	89.5	0.26	0.21	0.20
Jiangsu	441.3	112.1	44.9	42.6	21.3	6.4	-7.04	85.0	0.11	0.11	0.14
Jiangxi	1444.0	199.2	66.6	147.4	27.5	9.5	-10.14	93.6	0.35	0.13	0.12
Jilin	388.1	89.9	32.3	68.9	29.9	13.4	-6.07	80.6	0.24	0.20	0.24
Liaoning	253.9	55.7	31.1	43.8	24.1	11.2	-5.05	74.4	0.17	0.14	0.19
Macao	2.5	1.0	0.8	36.7	23.7	11.9	-4.18	67.6	1.04	0.62	0.66
Ningxia	168.0	37.9	12.4	122.6	34.1	11.6	-8.72	90.5	0.31	0.16	0.15
Qinghai	271.2	91.0	36.0	206.1	97.5	40.5	-6.03	80.3	0.52	0.39	0.41
Shaanxi	307.1	183.8	94.3	39.9	40.4	18.4	-2.87	53.9	0.10	0.21	0.32
Shandong	788.3	178.5	108.8	75.1	37.3	12.8	-6.55	83.0	0.15	0.15	0.30
Shanghai	62.7	14.80	13.7	31.1	7.1	5.3	-6.59	83.0	0.30	0.17	0.26
Shanxi	922.0	192.0	80.3	137.4	43.8	16.2	-7.91	88.2	0.44	0.31	0.33
Sichuan	2914.7	529.6	129.4	116.9	54.5	13.0	-8.13	88.9	0.24	0.22	0.18
Taiwan	57.5	26.2	26.7	17.6	12.6	15.2	-0.54	13.6	0.4	0.57	1.19
Tianjin	81.9	22.0	14.1	49.0	19.2	9.7	-6.01	80.2	0.35	0.32	0.44
Tibet	205.9	106.4	58.4	323.4	158.6	82.7	-5.05	74.4	0.38	0.25	0.25
Xinjiang	785.9	382.0	169.9	208.8	105.8	41.6	-5.98	80.1	0.69	0.86	0.91
Yunnan	1276.1	491.0	126.0	128.3	63.4	18.3	-7.21	85.7	0.25	0.22	0.18
Zhejiang	445.7	69.1	29.0	45.9	10.4	4.2	-8.89	90.8	0.14	0.08	0.12

*MMR* Maternal mortality ratio (number of maternal deaths per 100,000 livebirths), *SDI* Socio-demographic Index

**Table 2:** Number of maternal deaths and maternal mortality ratio in 1990 and 2017 of China, by age group

	**Number of maternal deaths**		**Maternal mortality ratio**	
**Age group (years)**	**1990**	**2017**	**1990**	**2017**
10 to 14	76.4 (66.1–88.3)	6.8 (6.0–7.7)	NA	NA
15 to 19	1465.3 (1270.3–1675)	69.8 (60.7.5–81)	167.4 (145.1–191.4)	21.7 (18.8–25.1)
20 to 24	5903.8 (5266.6–6564.7)	245 (214.4.0–284.1)	53.3 (47.5–59.3)	6.1 (5.3–7)
25 to 29	6091.7 (5492.8–6736.2)	663.2 (597.7.5–735.7)	73.4 (66.2–81.1)	9.3 (8.3–10.3)
30 to 34	4609.2 (4142.5–5032)	617.2 (553.7.0–697.2)	183.1 (164.6–199.9)	17.1 (15.3–19.3)
35 to 39	3061.9 (2695.4–3418.1)	330 (297.7.5–370.1)	356 (313.4–397.5)	34.3 (30.9–38.4)
40 to 44	1037.1 (906.9–1180.3)	174.5 (156.6.0–198.1)	1130(988.2–1286.1)	58.3 (52.4–66.2)
45 to 49	295.3 (264.3–331.3)	101.6 (90.4.5–113.8)	1062.9 (951.4–1192.5)	201.5 (179.3–225.8)
50 to 54	74.5 (66.3–82.2)	32.5 (28.9.0–36.4)	NA	NA

*NA* Not available

**Fig. 2:**
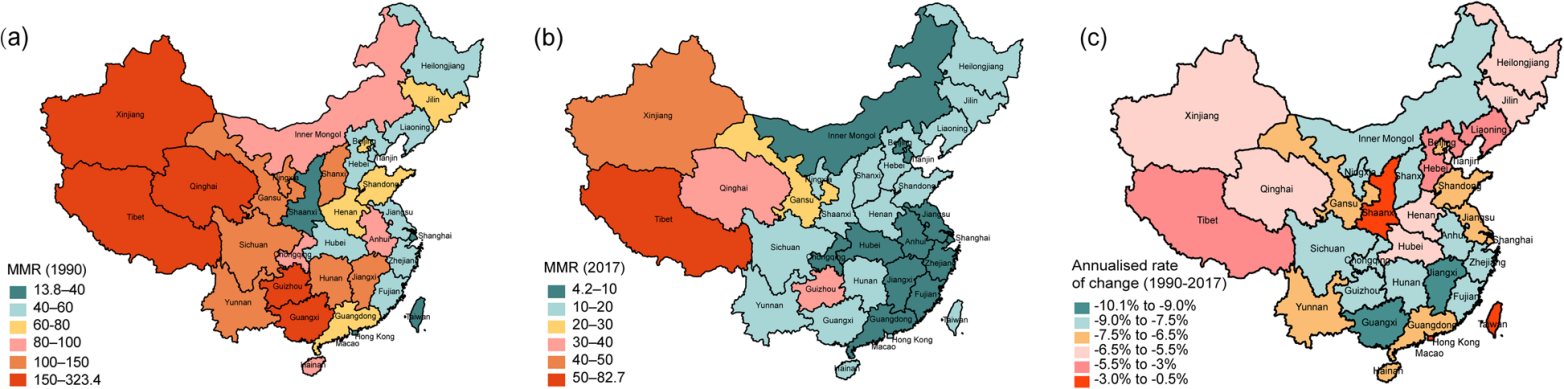
Maternal mortality ratio (MMR) at the province level in **a** 1990, **b** 2017, and **c** annualised rate of change in MMR from 1990 to 2017 in the provinces of China

In 1990, the MMR showed considerable heterogeneity across provinces in mainland China, ranged from 31.1 in Shanghai to 323.4 in Tibet (Table 1, Fig. 2a). Shanghai, Hebei, Jiangsu, Liaoning, Shaanxi, Tianjin and Zhejiang had MMR lower than 50 (Table 1, Fig. 2a).

We recorded remarkable decreases in MMR for all provinces up to 2017, especially in Jiangxi, the MMR in this province reduced from 147.4 in 1990 to 9.5 in 2017, decreased by 93.6%. Twenty-seven provinces achieved the MDG 5 target of a 75% reduction in MMR by 2017, and 30 provinces had MMR less than 20 (Table 1, Fig. 2b). In China, 11 provinces (Anhui, Tianjin, Guangdong, Jiangxi, Inner Mongolia, Beijing, Chongqing, Fujian, Jiangsu, Shanghai and Zhejiang) had MMR below this level. Tibet still had the highest MMR (82.7) in 2017; however, compared with 1990 it had reduced by 74.4%. All the remained provinces in China had MMR below the targeted Sustainable Development Goal (SDG) 3.1 level of 70.

**Fig. 3:**
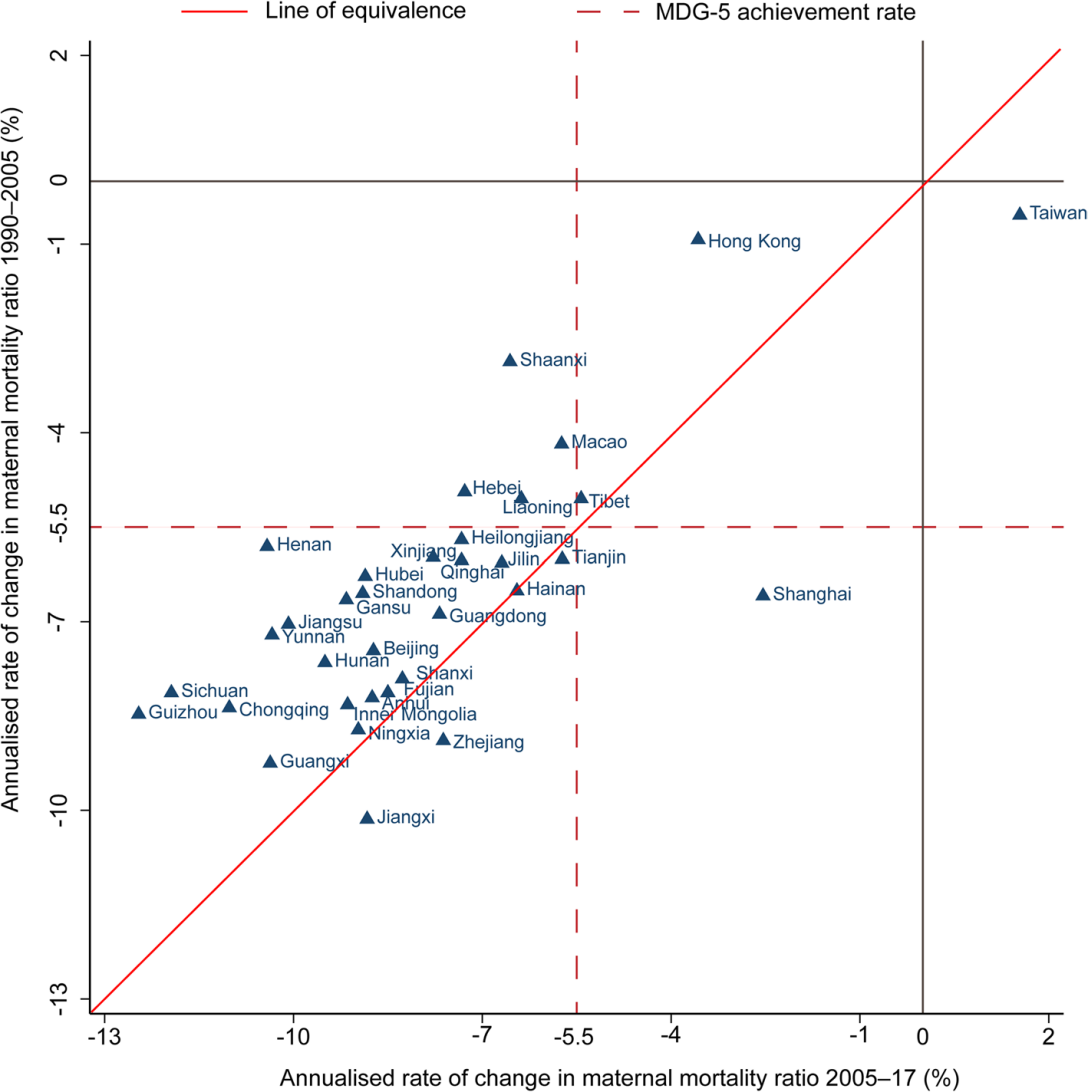
Province-level annualized rate of change in maternal mortality ratio from1990 to 2005, and 2005 to 2017. MDG-5, Millennium Development Goals 5

All provinces in China showed rapid decline in MMR by 2017. However, progress at the province level has been heterogeneous. The annualised rate of decline between 1990 and 2017 ranged from 10.14% in Jiangxi to 0.93% in Hong Kong SAR (Table 1, Fig. 2c). Twenty-eight provinces had higher annualised rates of decline in MMR between 2005 and 2017 than between 1990 and 2005 (Fig. 3). Thirty-two provinces had achieved the MDG 5 decrease of 5.5% per year in either time, except for Hong Kong SAR and Taiwan that already reached a low MMR since 1990. From 1990 to 2017, 27 provinces had reductions in MMR greater than 5.5% per year, except for Liaoning (-5.05%), Tibet (-5.05%), Hebei (-4.94%), Macao SAR (-4.18%), Shaanxi (-2.87%), Hong Kong SAR (-0.93%) and Taiwan (-0.54%) (Table 1).

In the province level, the observed maternal deaths was lower than expected values based on the SDI in all provinces between 1990 and 2017, except for Beijing, Xinjiang, Macao SAR and Taiwan at some point during the MDG period that had a higher observed than expected values. 30 provinces had observed maternal deaths at least 50% lower than expected in 2017 (Table 1, Fig. S1). These provinces had widely varying levels of social and economic development (Table S1). Thus, although lower observed than expected values are not surprising for the 14 high and high­middle SDI provinces with good public health intervention programmes, such as Jiangsu and Zhejiang, it is noteworthy that the remaining 16 provinces with lower levels of economic development, such as Guizhou and Ningxia, also achieved impressive improvement (Table 1, Fig. S1).

### Cause pattern of maternal mortality

**Fig. 4:**
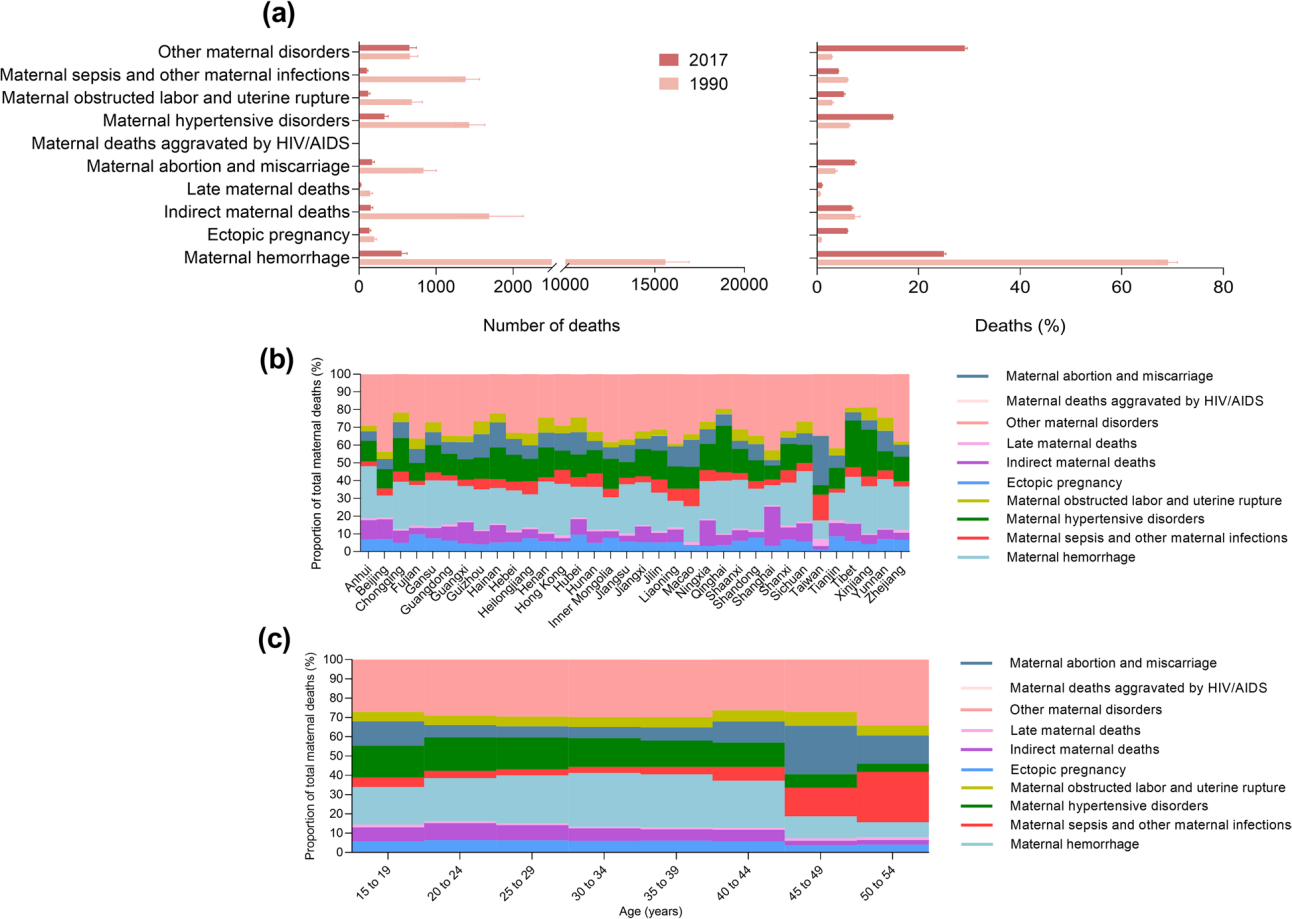
Causes of maternal death. **a** Total number (left) and mean percentage (right) of maternal deaths due to different causes in 1990 and 2017. Error bars show 95% uncertainty intervals. **b** Percentage of maternal deaths due to different causes in 2017, by province. **c** Percentage of maternal deaths due to different causes in 2017, by age group

In 1990, haemorrhage, indirect maternal causes, and hypertensive disorders were the top three causes for maternal deaths in China. Notably, haemorrhage was the leading specific cause in all provinces (Table S2). Nationally, it accounted for 69.1% (15,595.5/22615.2) of all maternal deaths that is far greater than the other causes (Fig. 4a). Except for other maternal disorders and deaths aggravated by HIV/AIDS, the absolute numbers of maternal deaths due to all other causes decreased significantly from 1990 to 2017 (Fig. 4a, Fig. S2). The greatest reduction was seen in deaths due to maternal haemorrhage: from 15,595.5 (95% UI 14,341.6–16,922.7) in 1990, to 553.6 (95% UI 493.3–625.2) in 2017, decreased by 96.5% (Fig. 4a). In 2017, except for other non-specific maternal disorders, haemorrhage and hypertensive disorders were still the top two leading causes for maternal mortality in China, accounted for 553.6 (95% UI 493.3–625.2) and 331.8 (95% UI 291.6–378.1) deaths respectively (Fig. 4a, Table S3).

The biggest percentage decrease was also seen in maternal haemorrhage, which caused 69.1% (95% UI 67.2–70.9%) of all maternal deaths in 1990, but dropped to 25.0% (95% UI 24.6–25.4%) in 2017. The percentage of maternal deaths due to ectopic pregnancy, abortion and miscarriage, hypertensive disorders, obstructed labor and uterine rupture, and late maternal deaths increased from 1990 to 2017 (Fig. 4a, Fig. S3). Except for other non-specific disorders, haemorrhage and hypertensive disorders were the top two leading causes that accounted for the highest proportion of maternal deaths in 2017 (Fig. 4a). The top 5 provinces with highest proportion of maternal deaths due to haemorrhage were Qinghai (29.9%), Anhui (29.4%), Hong Kong SAR (28.8%), Sichuan (28.6%) and Henan (28.3%); the top 5 provinces with highest proportion of maternal deaths due to hypertensive disorders were Xinjiang (26.5%), Tibet (26.3%), Qinghai (26.2%), Chongqing (19.1%) and Hainan (18.0%) (Fig. 4b).

Cause patterns vary by age. The proportion of maternal deaths caused by haemorrhage increased with age in 15–34 years group, and falling from a peak of 27.9% in 30–34 years group to 7.8% in 50–54 years group. The proportion of abortion and miscarriage caused death was at low level in women aged 20–39 years, ranged from 5.7% to 6.8%. However, it raised sharply to 25.1% in women aged 45–49 years. Hypertensive disorders caused maternal deaths were more commonly observed in young mothers, which accounted for 17.4% of total maternal deaths in women aged 20–24 years and reduced with advancing age, dropped to 4.3% in women aged 45–49 years. In contrary, maternal deaths caused by sepsis and other infections showed rapid increase with advancing age, ranged from 3.2% to 5.1% in women younger than 40 years, but reached 26.1% in 50–54 years group (Fig. 4c). The provincial number of maternal deaths by cause in each age group in 2017 are presented in Table S4.

## Discussion

In China, there are more than 17 million livebirths each year [12]. Based on the data sources from GBD 2017, we describe the level, trends and cause patterns of the MMR for each province over a nearly 30-year period. We found substantial variation in MMR across provinces, but most (27/34) showed significant declines greater than the MDG 5 target of 5.5% per year during 1990–2017, and 33 of the 34 provinces have achieved the MMR below the SDG 3.1 target level of 70 in 2017. This systematic analysis uncovers that haemorrhage and hypertensive disorders are the top two specific causes of maternal death currently in China, followed by abortion and miscarriage, indirect causes, and ectopic pregnancy. Mothers older than 40 years have a much higher MMR than those aged 20–29 years.

The drivers of improvement for MMR reduction are variable and multifaceted, including clinical skill, public health and national policy implications. Higher SDI and improved economic conditions usually contribute substantially to decline in MMR [5]. The lowest provincial MMR level in 2017 was 4.2 in Zhejiang, which was similar to those in the most developed countries, including Austria (4.3), Ireland (3.9), Finland (3.6), Italy (3.6), and Denmark (3.6) (https://maternalhealthatlas.org/). However, income per capita in 1996 of China could explain only about 10% of the variation in the annualised rate of decline in MMR over the next two decades; in addition, from 1996 to 2015 the income improvement could explain only about 18% of the changes in MMR [4]. It should be noted that the number of deaths and reductions in MMR need to be considered together to interpret the trends of maternal mortality. For example, meaning and reason for low reduction in MMR for Hong Kong SAR and Tibet are different. It is difficult to achieve a high decline when the number of deaths is already low.

The lower MMR might partly be attributed to the lower fertility rates, but seems more likely to result from interventions introduced by the national Reducing Maternal Mortality and Eliminating Neonatal Tetanus programme of China since 2000. In order to eliminate the inequality of MMR between economically rich and underdeveloped areas, the Chinese government launched this national programme to improve the maternal and neonatal health. It was initially administered to cover the midwestern regions of China, but had become a national policy by 2009. Of this policy, some objectives should be noted, for they might provide effective reference for public health policy makers in other countries (particularly in those with lower economic levels) to devise effective plans for improving maternal health. These objectives consist of: improve obstetric health care at the county, township, and village levels; establish obstetric emergency centres and develop a green channel for obstetric intensive care services in all 2852 counties of China; improve the proportion of in-hospital deliveries and provide financial support for women in rural areas and with low incomes; improve maternal health education; and strengthen supervision of obstetric health care in all medical institutions [4].

In addition, due to the rapid economic development in the past two decades, comprehensive sex education, methods of modern contraception and access to safe abortion might all have contributed to the progress in maternal health and the reduction in maternal mortality. Increased coverage of high-quality antenatal care, and effective identifying and treatment of infectious diseases (e.g., chronic hepatitis B infection), pre-existing chronic conditions (e.g., renal dysfunction, rheumatic, or heart disease) and pregnancy abnormalities (e.g., nutritional deficiencies, hypertensive disorder, and hyperglycemia) are also essential for prevention of maternal deaths [4, 13, 14].

From 2010 to 2015, the percentage of in-hospital delivery increased from 96.3% to 99.7%. During the same period, substantial efforts were also made to offer systematic care for mothers from before to after delivery, and the percentage of pregnancies received these services increased from 80.9% to 91.5% [12]. Despite these impressive improvements, our results showed that five provinces still had MMR significantly higher than the national mean level of 13.6 in 2017, including Tibet (82.7), Xinjiang (41.6), Qinghai (40.5), Guizhou (30.9) and Gansu (23.0). Affected by traditional customs, women in these regions prefer to give birth at home [15]. Besides, low levels of education or even illiteracy have also limited the ability of pregnant women to seek the provision of formal healthcare services [16]. Excluding the educational level, the maternal healthcare medical resources in these areas are also at a disadvantage. Information from the China Health and Family Planning Statistical Year book for 2014 (http://tongji.cnki.net/kns55/navi/YearBook.aspx?id=N2014120147&floor=1) revealed that the number of personnel maternal healthcare service per 1,000 persons for the five provinces was lower than other regions in China. The central and local governments should make a concerted effort and devise appropriate interventions to help these provinces. To sum up, cultural practices and low in-hospital delivery rate were reported as major factors behind the high MMR in the western and southwestern areas [13, 14, 17]. Tough natural environments, weaker health services and difficulty in accessing convenient transportation might also contribute to the high MMR in these provinces [4]. Increased coverage of access to and quality of health care could contribute to further declines in maternal mortality in China.

Previous report revealed that more than 95% of maternal deaths in western regions and 76% in eastern regions of China are preventable [13]. Haemorrhage was the leading specific cause of maternal deaths across the provinces in China, accounted for about 25% of all maternal deaths in 2017. It was reported that with advancing in sufficient blood supply, skilful medical procedures and easy transportation, 90% of obstetric haemorrhage caused deaths would be preventable [18]. Continued promotion of policies to control pregnancy-induced hypertension, reduce anaemia and malnutrition, encourage skilled birth attendance and in-hospital delivery, discourage adolescent fertility, and prevent unsafe abortion should lead to sustained maternal health improvement [19–21]. In addition, special obstetric care focused on fatal conditions of the peripartum and late maternal period, such as pulmonary embolism, cardiomyopathy, and renal complications are also needed to be improved to reduce the preventable maternal deaths. Enhanced data collection with more detailed information on these interventions at the province or even county level will be necessary in the future, which can help the government to create policies with precision.

This study provides comprehensive estimates of the levels and trends of maternal mortality due to different causes, by age and province for the period 1990 to 2017 in China. The limitations of our study should also be pointed out. First, because of sparse data, only nine specific causal categories of maternal death were examined in this report. We were unable to quantify the contributions of other conditions, such as obesity, diabetes, pulmonary embolism, cardiac disorders and hepatitis to the maternal mortality. Second, other potential data sources on maternal health, such as the ARMCH, and the Disease Surveillance Point system administered by the Chinese Centre for Disease Control and Prevention, are key sources of vital statistics for maternal mortality. These data are important for the accurate estimation of levels and trends in maternal mortality of China. However, they did not provide detailed information on cause­specific MMR estimates and the age groups of women. As civil registration systems provide essential information for public health policy devising and disease preventions [22], strengthening of these systems is vital for public health. Third, determination on which deaths of pregnant women should be defined as maternal deaths may influence the diagnosis and estimates of maternal mortality. For example, incidental deaths in which pregnancy had no causal role might be misclassified as maternal deaths. Additionally, in the current stage, China uses all possible medical resources to lower the risk of maternal mortality during pregnancy or within 42 days of termination of pregnancy. Insufficient attention on late maternal mortality may lead to under-estimation of the maternal deaths.

## Conclusions

In summary, substantial progress in reducing MMR has been achieved in China during the last two decades, but there is still major work left to do, for disparities in MMR remained striking across provinces. More than 2240 women died during or following pregnancy in 2017, most of which might be preventable deaths. Haemorrhage and hypertensive disorders are the main drivers of maternal death that need intensified focus. Setting associated interventions will need careful consideration of regions that still have MMR significantly higher than the national mean level, such as western China.

## Supplementary Information

**Additional file 1: Table S1.** SDI values and quintile groupings for China, 2017. **Table S2.** Provincial number of maternal deaths by cause in 1990. **Table S3.** Provincial number of maternal deaths by cause in 2017. **Table S4.** Provincial number of maternal deaths by cause in each age group in 2017. **Figure S1.** Observed maternal deaths, and expected maternal deaths probabilistically predicted based on the Socio-demographic Index for the 34 provinces, from 1990 to 2017. **Figure S2.** Cause and number of maternal deaths from 1990 to 2017 in China. **Figure S3.** Cause and percentage of maternal deaths from 1990 to 2017 in China.

## Data Availability

The datasets are available from the Maternal Health Atlas of GBD 2017 (publicly available online: https://maternalhealthatlas.org/). The data that support the findings of this study are available from the corresponding author upon reasonable request.

## Abbreviations

ARC: Annualized rate of change
GATHER: Guidelines for Accurate and Transparent Health Estimates Reporting
GBD: Global Burden of Diseases
MDG: Millennium Development Goals
MMR: Maternal mortality ratio
SDI: Socio-demographic Index
UI: Uncertainty intervals

## References

[1] United Nations (2013). United Nations Millennium Development Goals.

[2] United Nations’ Secretary General (2010). Global Strategy for Women’s and Children’s Health.

[3] Kassebaum NJ, Barber RM, Bhutta ZA, Dandona L, Gething PW, Hay SI (2016). Global, regional, and national levels of maternal mortality, 1990–2015: a systematic analysis for the Global Burden of Disease Study 2015. Lancet.

[4] Liang J, Li X, Kang C, Wang Y, Kulikoff XR, Coates MM (2019). Maternal mortality ratios in 2852 Chinese counties, 1996–2015, and achievement of Millennium Development Goal 5 in China: a subnational analysis of the Global Burden of Disease Study 2016. Lancet.

[5] GBD 2015 Maternal Mortality Collaborators (2016). Global, regional, and national levels of maternal mortality, 1990–2015: a systematic analysis for the Global Burden of Disease Study 2015. Lancet.

[6] Kassebaum NJ, Bertozzi-Villa A, Coggeshall MS, Shackelford KA, Steiner C, Heuton KR (2014). Global, regional, and national levels and causes of maternal mortality during 1990–2013: a systematic analysis for the Global Burden of Disease Study 2013. Lancet.

[7] Ruppert D, Matteson DS (2015). Return calculations. Statistics and Data Analysis for Financial Engineering.

[8] Murray CJL, Callender CSKH, Kulikoff XR, Srinivasan V, Abate D, Abate KH (2018). Population and fertility by age and sex for 195 countries and territories, 1950–2017: a systematic analysis for the Global Burden of Disease Study 2017. Lancet.

[9] Dicker D, Nguyen G, Abate D, Abate KH, Abay SM, Abbafati C (2018). Global, regional, and national age-sex-specific mortality and life expectancy, 1950–2017: a systematic analysis for the Global Burden of Disease Study 2017. Lancet.

[10] Kyu HH, Abate D, Abate KH, Abay SM, Abbafati C, Abbasi N (2018). Global, regional, and national disability-adjusted life-years (DALYs) for 359 diseases and injuries and healthy life expectancy (HALE) for 195 countries and territories, 1990–2017: a systematic analysis for the Global Burden of Disease Study 2017. Lancet.

[11] Stevens GA, Alkema L, Black RE, Boerma JT, Collins GS, Ezzati M (2016). Guidelines for Accurate and Transparent Health Estimates Reporting: the GATHER statement. Lancet.

[12] DESA (2017). INT/1: interpolated demographic indicators by region, subregion and country, annually for 1950–2099. World population prospects: the 2017 revision.

[13] Liang J, Dai L, Zhu J, Li X, Zeng W, Wang H (2011). Preventable maternal mortality: geographic/rural-urban differences and associated factors from the population-based Maternal Mortality Surveillance System in China. BMC Public Health.

[14] Li J, Liang J, Wang J, Ren Z, Yang D, Wang Y (2020). Spatiotemporal trends and ecological determinants in maternal mortality ratios in 2,205 Chinese counties, 2010–2013: A Bayesian modelling analysis. Plos Med.

[15] Song P, Kang C, Theodoratou E, Rowa-Dewar N, Liu X, An L (2016). Barriers to Hospital Deliveries among Ethnic Minority Women with Religious Beliefs in China: A Descriptive Study Using Interviews and Survey Data. Int J Environ Res Public Health.

[16] Mirowsky J, Ross CE (2003). Education, social status, and health.

[17] Du Q, Nass O, Bergsjo P, Kumar BN (2009). Determinants for high maternal mortality in multiethnic populations in western China. Health Care Women Int.

[18] Berg CJ, Harper MA, Atkinson SM, Bell EA, Brown HL, Hage ML (2005). Preventability of pregnancy-related deaths: results of a state-wide review. Obstet Gynecol.

[19] Konje JC, Ladipo OA (2000). Nutrition and obstructed labor. Am J Clin Nutr.

[20] Bhutta ZA, Das JK, Rizvi A, Gaffey MF, Walker N, Horton S (2013). Evidence-based interventions for improvement of maternal and child nutrition: what can be done and at what cost?. Lancet.

[21] Bhutta ZA, Ahmed T, Black RE, Cousens S, Dewey K, Giugliani E (2008). What works? Interventions for maternal and child undernutrition and survival. Lancet.

[22] Phillips DE, AbouZahr C, Lopez AD, Mikkelsen L, de Savigny D, Lozano R (2015). Are well functioning civil registration and vital statistics systems associated with better health outcomes?. Lancet.

