# Supplementary material for: The trends of maternal mortality ratios and cause pattern in 34 Chinese provinces, 1990–2017
Source: BMC Public Health. 2022 Jul 16;22:1369. doi: 10.1186/s12889-022-13770-0 (PMC9288211; doi:10.1186/s12889-022-13770-0)

**Table S1** SDI values and quintile groupings for China, 2017

| Location | SDI Quintile | SDI value |
| --- | --- | --- |
| China | High-middle SDI | 0.71 |
| Anhui | Middle SDI | 0.62 |
| Beijing | High SDI | 0.85 |
| Chongqing | Middle SDI | 0.66 |
| Fujian | High-middle SDI | 0.71 |
| Gansu | Low-middle SDI | 0.6 |
| Guangdong | High-middle SDI | 0.76 |
| Guangxi | Middle SDI | 0.68 |
| Guizhou | Low-middle SDI | 0.57 |
| Hainan | Middle SDI | 0.68 |
| Hebei | High-middle SDI | 0.7 |
| Heilongjiang | High-middle SDI | 0.7 |
| Henan | Middle SDI | 0.68 |
| Hong Kong | High SDI | 0.82 |
| Hubei | Middle SDI | 0.69 |
| Hunan | Middle SDI | 0.68 |
| Inner Mongolia | High-middle SDI | 0.71 |
| Jiangsu | High-middle SDI | 0.74 |
| Jiangxi | Middle SDI | 0.64 |
| Jilin | High-middle SDI | 0.71 |
| Liaoning | High-middle SDI | 0.74 |
| Macao | High SDI | 0.83 |
| Ningxia | Middle SDI | 0.64 |
| Qinghai | Middle SDI | 0.62 |
| Shaanxi | Middle SDI | 0.68 |
| Shandong | High-middle SDI | 0.74 |
| Shanghai | High SDI | 0.82 |
| Shanxi | Middle SDI | 0.7 |
| Sichuan | Middle SDI | 0.66 |
| Taiwan | High-middle SDI | 0.86 |
| Tianjin | High-middle SDI | 0.8 |
| Tibet | Low-middle SDI | 0.47 |
| Xinjiang | Middle SDI | 0.68 |
| Yunnan | Middle SDI | 0.62 |
| Zhejiang | High-middle SDI | 0.74 |

SDI, Socio-demographic Index.

**Table S2** Provincial number of maternal deaths by cause in 1990

| Province | Cause of maternal deaths | | | | | | | | | |
| --- | --- | --- | --- | --- | --- | --- | --- | --- | --- | --- |
|  | Ectopic pregnancy | Indirect maternal deaths | Late maternal deaths | Maternal abortion and miscarriage | Maternal deaths aggravated by HIV/AIDS | Maternal hemorrhage | Maternal hypertensive disorders | Maternal obstructed labor and uterine rupture | Maternal sepsis and other maternal infections | Other maternal disorders |
| China overall | 197.8 | 1688.4 | 144.5 | 835.4 | 0.7 | 15595.5 | 1428.1 | 684.9 | 1381.4 | 658.5 |
| Anhui | 31.6 | 194.5 | 9.0 | 18.4 | 0.0 | 905.8 | 92.4 | 33.3 | 19.6 | 62.3 |
| Hainan | 1.2 | 14.6 | 0.8 | 16.2 | 0.0 | 66.8 | 14.6 | 6.4 | 13.3 | 2.4 |
| Hebei | 6.5 | 32.8 | 4.7 | 29.2 | 0.0 | 394.3 | 69.0 | 13.2 | 53.8 | 16.5 |
| Heilongjiang | 3.4 | 11.1 | 2.4 | 25.1 | 0.0 | 188.5 | 21.8 | 18.9 | 46.2 | 5.9 |
| Henan | 10.6 | 47.6 | 9.0 | 102.1 | 0.1 | 901.5 | 126.5 | 95.0 | 56.3 | 28.7 |
| Hong Kong | 0.1 | 0.2 | 0.2 | 2.0 | 0.0 | 2.9 | 0.5 | 0.6 | 2.6 | 0.3 |
| Hubei | 10.7 | 46.2 | 3.6 | 16.9 | 0.0 | 359.6 | 40.5 | 55.7 | 17.0 | 14.6 |
| Hunan | 5.7 | 53.7 | 7.8 | 34.7 | 0.1 | 887.9 | 72.6 | 34.3 | 164.6 | 25.9 |
| Inner Mongolia | 4.6 | 16.3 | 2.8 | 15.4 | 0.0 | 298.5 | 39.6 | 8.9 | 37.0 | 9.7 |
| Jiangsu | 5.4 | 32.3 | 3.7 | 49.2 | 0.0 | 243.9 | 35.0 | 17.2 | 42.5 | 12.0 |
| Jiangxi | 9.9 | 122.5 | 8.4 | 14.5 | 0.0 | 1079.8 | 80.7 | 17.1 | 23.5 | 87.6 |
| Beijing | 1.0 | 30.1 | 1.0 | 10.2 | 0.0 | 33.1 | 10.0 | 7.4 | 14.1 | 5.9 |
| Jilin | 2.6 | 19.9 | 2.7 | 27.9 | 0.0 | 229.1 | 32.9 | 9.1 | 59.6 | 4.2 |
| Liaoning | 2.2 | 21.3 | 2.3 | 41.6 | 0.0 | 117.1 | 22.5 | 4.1 | 34.0 | 8.7 |
| Macao | 0.1 | 0.0 | 0.0 | 0.4 | 0.0 | 0.5 | 0.4 | 0.1 | 0.7 | 0.2 |
| Ningxia | 0.5 | 14.0 | 1.0 | 2.7 | 0.0 | 123.5 | 10.1 | 1.9 | 12.2 | 1.9 |
| Qinghai | 1.9 | 10.8 | 1.5 | 7.7 | 0.0 | 195.0 | 28.9 | 3.7 | 18.4 | 3.4 |
| Shaanxi | 2.5 | 10.4 | 1.8 | 7.0 | 0.0 | 232.0 | 17.8 | 8.0 | 22.0 | 5.7 |
| Shandong | 8.1 | 44.0 | 6.5 | 49.1 | 0.0 | 452.7 | 81.0 | 39.3 | 79.9 | 27.7 |
| Shanghai | 0.5 | 22.7 | 0.6 | 2.7 | 0.0 | 16.7 | 5.0 | 5.2 | 7.3 | 1.9 |
| Shanxi | 9.7 | 81.5 | 6.6 | 4.5 | 0.0 | 610.0 | 85.8 | 18.8 | 43.4 | 61.7 |
| Sichuan | 6.6 | 280.7 | 16.9 | 10.8 | 0.1 | 2339.0 | 62.5 | 49.2 | 91.8 | 57.1 |
| Chongqing | 2.9 | 11.5 | 2.0 | 12.9 | 0.0 | 238.5 | 17.4 | 7.1 | 24.6 | 2.8 |
| Tianjin | 0.9 | 12.4 | 1.0 | 8.4 | 0.0 | 37.8 | 7.1 | 4.1 | 6.2 | 4.0 |
| Tibet | 1.8 | 13.5 | 1.1 | 6.7 | 0.0 | 137.9 | 22.7 | 3.3 | 16.7 | 2.1 |
| Xinjiang | 5.8 | 22.8 | 4.3 | 19.2 | 0.1 | 555.3 | 67.5 | 23.1 | 79.7 | 8.2 |
| Yunnan | 8.2 | 33.8 | 7.0 | 98.1 | 0.1 | 968.3 | 35.3 | 34.5 | 77.9 | 13.0 |
| Zhejiang | 7.7 | 44.9 | 3.9 | 49.5 | 0.0 | 220.3 | 66.5 | 11.1 | 24.1 | 17.7 |
| Fujian | 4.4 | 16.0 | 3.0 | 31.3 | 0.0 | 314.1 | 21.9 | 19.9 | 22.6 | 10.3 |
| Gansu | 8.2 | 24.8 | 5.1 | 24.2 | 0.0 | 675.3 | 50.7 | 24.7 | 58.8 | 12.7 |
| Guangdong | 8.7 | 74.9 | 6.4 | 65.7 | 0.0 | 584.3 | 57.7 | 20.1 | 58.1 | 21.9 |
| Guangxi | 14.5 | 211.8 | 7.6 | 8.0 | 0.1 | 850.6 | 49.6 | 40.1 | 70.8 | 61.1 |
| Guizhou | 9.4 | 114.7 | 9.5 | 23.1 | 0.1 | 1334.7 | 81.6 | 49.5 | 82.0 | 60.5 |
| Guizhou | 9.4 | 114.7 | 9.5 | 23.1 | 0.1 | 1334.7 | 81.6 | 49.5 | 82.0 | 60.5 |

**Table S3** Provincial number of maternal deaths by cause in 2017

| Location | Ectopic pregnancy | Indirect maternal deaths | Late maternal deaths | Maternal abortion and miscarriage | Maternal deaths aggravated by HIV/AIDS | Maternal hemorrhage | Maternal hypertensive disorders | Maternal obstructed labor and uterine rupture | Maternal sepsis and other maternal infections | Other maternal disorders |
| --- | --- | --- | --- | --- | --- | --- | --- | --- | --- | --- |
| China Overall | 134.3 | 152.3 | 21.9 | 170.4 | 0.4 | 553.6 | 331.8 | 119.3 | 102.2 | 654.3 |
| Anhui | 6.0 | 9.1 | 0.8 | 4.5 | 0.0 | 25.0 | 9.9 | 2.6 | 2.3 | 24.8 |
| Beijing | 1.6 | 2.5 | 0.2 | 1.3 | 0.0 | 2.8 | 2.5 | 0.9 | 0.9 | 10.0 |
| Chongqing | 1.5 | 2.0 | 0.3 | 2.7 | 0.0 | 8.0 | 5.7 | 1.6 | 1.7 | 6.5 |
| Fujian | 3.8 | 1.4 | 0.5 | 3.0 | 0.0 | 9.0 | 3.9 | 2.3 | 0.9 | 14.1 |
| Gansu | 6.8 | 5.2 | 0.7 | 6.5 | 0.0 | 23.5 | 13.8 | 5.0 | 4.1 | 24.4 |
| Guangdong | 10.4 | 12.7 | 2.2 | 10.8 | 0.0 | 40.4 | 20.0 | 5.9 | 4.9 | 56.9 |
| Guangxi | 4.1 | 11.0 | 0.8 | 8.8 | 0.1 | 17.9 | 10.6 | 3.1 | 3.3 | 31.9 |
| Guizhou | 4.2 | 7.6 | 0.6 | 13.4 | 0.0 | 23.4 | 12.2 | 7.5 | 6.2 | 27.1 |
| Hainan | 1.1 | 2.1 | 0.2 | 2.9 | 0.0 | 4.2 | 3.8 | 1.1 | 1.0 | 4.6 |
| Hebei | 7.1 | 7.2 | 1.5 | 11.6 | 0.0 | 29.6 | 20.0 | 4.6 | 6.5 | 43.4 |
| Heilongjiang | 2.0 | 1.3 | 0.3 | 2.0 | 0.0 | 4.9 | 3.3 | 1.7 | 1.9 | 8.8 |
| Henan | 11.7 | 8.7 | 2.1 | 16.7 | 0.0 | 57.4 | 34.2 | 17.0 | 5.0 | 49.9 |
| Hong Kong | 0.3 | 0.1 | 0.1 | 0.6 | 0.0 | 1.8 | 0.7 | 0.3 | 0.5 | 1.8 |
| Hubei | 5.0 | 4.6 | 0.6 | 6.6 | 0.0 | 9.1 | 6.8 | 4.4 | 2.8 | 12.8 |
| Hunan | 5.1 | 6.6 | 1.0 | 5.4 | 0.0 | 24.9 | 13.4 | 5.2 | 7.8 | 33.4 |
| Inner Mongolia | 2.0 | 0.9 | 0.3 | 1.6 | 0.0 | 4.6 | 4.3 | 0.7 | 1.2 | 9.7 |
| Jiangsu | 2.5 | 1.5 | 0.7 | 4.3 | 0.0 | 12.3 | 4.0 | 1.5 | 1.7 | 16.5 |
| Jiangxi | 3.5 | 6.0 | 0.6 | 3.8 | 0.0 | 16.0 | 10.3 | 2.8 | 2.2 | 21.5 |
| Jilin | 1.6 | 1.8 | 0.4 | 2.7 | 0.0 | 7.0 | 5.3 | 1.1 | 2.4 | 10.1 |
| Liaoning | 1.7 | 2.2 | 0.4 | 3.5 | 0.0 | 4.6 | 3.9 | 0.4 | 2.2 | 12.3 |
| Macao | 0.0 | 0.0 | 0.0 | 0.1 | 0.0 | 0.2 | 0.1 | 0.0 | 0.1 | 0.3 |
| Ningxia | 0.4 | 1.8 | 0.1 | 1.0 | 0.0 | 2.6 | 1.8 | 0.5 | 0.8 | 3.3 |
| Qinghai | 1.4 | 2.1 | 0.2 | 2.3 | 0.0 | 10.8 | 9.4 | 1.1 | 1.8 | 7.0 |
| Shaanxi | 5.8 | 5.6 | 0.9 | 4.2 | 0.0 | 25.8 | 13.0 | 6.1 | 3.6 | 29.3 |
| Shandong | 8.5 | 3.6 | 1.4 | 9.8 | 0.0 | 25.1 | 12.8 | 5.3 | 4.7 | 37.7 |
| Shanghai | 0.4 | 3.0 | 0.1 | 0.4 | 0.0 | 1.5 | 1.1 | 0.8 | 0.5 | 5.9 |
| Shanxi | 5.6 | 5.3 | 0.9 | 2.9 | 0.0 | 19.4 | 11.9 | 3.1 | 5.6 | 25.6 |
| Sichuan | 7.2 | 13.4 | 1.2 | 8.3 | 0.0 | 37.0 | 13.5 | 8.6 | 5.9 | 34.5 |
| Taiwan | 0.3 | 0.5 | 1.1 | 7.4 | 0.0 | 2.8 | 1.4 | 0.1 | 3.9 | 9.2 |
| Tianjin | 1.2 | 1.1 | 0.2 | 1.0 | 0.0 | 2.2 | 1.7 | 0.6 | 0.3 | 5.9 |
| Tibet | 3.5 | 5.7 | 0.3 | 2.6 | 0.0 | 15.1 | 15.4 | 1.6 | 3.2 | 11.0 |
| Xinjiang | 7.4 | 8.5 | 1.2 | 8.7 | 0.1 | 45.5 | 44.9 | 12.4 | 9.4 | 31.8 |
| Yunnan | 8.9 | 6.7 | 0.9 | 14.5 | 0.1 | 35.0 | 13.6 | 9.2 | 6.3 | 31.0 |
| Zhejiang | 1.9 | 1.2 | 0.4 | 1.9 | 0.0 | 7.1 | 4.0 | 0.5 | 0.8 | 11.0 |

**Table S4** Provincial number of maternal deaths by cause in each age group in 2017

| Province and age group | Cause of maternal deaths | | | | | | | | | |
| --- | --- | --- | --- | --- | --- | --- | --- | --- | --- | --- |
|  | Ectopic pregnancy | Indirect maternal deaths | Late maternal deaths | Maternal abortion and miscarriage | Maternal deaths aggravated by HIV/AIDS | Maternal hemorrhage | Maternal hypertensive disorders | Maternal obstructed labor and uterine rupture | Maternal sepsis and other maternal infection | Other maternal disorders |
| **Anhui** |  |  |  |  |  |  |  |  |  |  |
| 10 to 14 | 0.01 | 0.02 | 0.01 | 0.02 | 0 | 0.03 | 0.01 | 0.01 | 0 | 0.08 |
| 15 to 19 | 0.1 | 0.17 | 0.02 | 0.12 | 0 | 0.3 | 0.18 | 0.04 | 0.04 | 0.41 |
| 20 to 24 | 0.71 | 1.29 | 0.1 | 0.41 | 0 | 2.48 | 1.29 | 0.24 | 0.2 | 2.77 |
| 25 to 29 | 2.03 | 3.66 | 0.27 | 1.2 | 0 | 8.72 | 3.77 | 0.79 | 0.54 | 8.43 |
| 30 to 34 | 1.48 | 2.14 | 0.19 | 0.88 | 0 | 7.32 | 2.48 | 0.67 | 0.38 | 6.36 |
| 35 to 39 | 0.82 | 1.05 | 0.11 | 0.52 | 0 | 3.74 | 1.19 | 0.36 | 0.24 | 3.29 |
| 40 to 44 | 0.43 | 0.6 | 0.06 | 0.46 | 0 | 1.65 | 0.57 | 0.2 | 0.21 | 1.52 |
| 45 to 49 | 0.22 | 0.14 | 0.05 | 0.7 | 0 | 0.54 | 0.31 | 0.21 | 0.42 | 1.32 |
| 50 to 54 | 0.15 | 0.07 | 0.02 | 0.18 | 0 | 0.2 | 0.09 | 0.08 | 0.27 | 0.59 |
| **Beijing** |  |  |  |  |  |  |  |  |  |  |
| 10 to 14 | 0 | 0 | 0 | 0 | 0 | 0 | 0 | 0 | 0 | 0.02 |
| 15 to 19 | 0.02 | 0.03 | 0 | 0.02 | 0 | 0.02 | 0.03 | 0.01 | 0.01 | 0.1 |
| 20 to 24 | 0.15 | 0.26 | 0.02 | 0.09 | 0 | 0.22 | 0.23 | 0.06 | 0.06 | 0.82 |
| 25 to 29 | 0.51 | 0.89 | 0.07 | 0.31 | 0 | 0.84 | 0.82 | 0.29 | 0.21 | 2.98 |
| 30 to 34 | 0.58 | 0.87 | 0.07 | 0.37 | 0 | 1.07 | 0.89 | 0.33 | 0.25 | 3.58 |
| 35 to 39 | 0.26 | 0.34 | 0.03 | 0.2 | 0 | 0.48 | 0.37 | 0.15 | 0.14 | 1.62 |
| 40 to 44 | 0.08 | 0.11 | 0.01 | 0.13 | 0 | 0.14 | 0.13 | 0.06 | 0.11 | 0.52 |
| 45 to 49 | 0.02 | 0.02 | 0.01 | 0.16 | 0 | 0.04 | 0.03 | 0.03 | 0.1 | 0.21 |
| 50 to 54 | 0 | 0.01 | 0 | 0.02 | 0 | 0 | 0 | 0 | 0.03 | 0.1 |
| **Chongqing** |  |  |  |  |  |  |  |  |  |  |
| 10 to 14 | 0 | 0 | 0 | 0.01 | 0 | 0.01 | 0.01 | 0.01 | 0 | 0.02 |
| 15 to 19 | 0.07 | 0.08 | 0.02 | 0.16 | 0 | 0.24 | 0.26 | 0.05 | 0.07 | 0.25 |
| 20 to 24 | 0.25 | 0.37 | 0.05 | 0.31 | 0 | 1.14 | 0.96 | 0.23 | 0.18 | 0.94 |
| 25 to 29 | 0.47 | 0.68 | 0.08 | 0.57 | 0 | 2.51 | 1.84 | 0.53 | 0.33 | 1.79 |
| 30 to 34 | 0.35 | 0.43 | 0.06 | 0.46 | 0 | 2.17 | 1.32 | 0.31 | 0.27 | 1.44 |
| 35 to 39 | 0.19 | 0.21 | 0.03 | 0.27 | 0 | 1.07 | 0.69 | 0.19 | 0.19 | 0.82 |
| 40 to 44 | 0.13 | 0.14 | 0.03 | 0.34 | 0 | 0.64 | 0.51 | 0.17 | 0.3 | 0.58 |
| 45 to 49 | 0.04 | 0.1 | 0.02 | 0.49 | 0 | 0.17 | 0.12 | 0.08 | 0.25 | 0.43 |
| 50 to 54 | 0 | 0.01 | 0.01 | 0.05 | 0 | 0.03 | 0.02 | 0.01 | 0.1 | 0.23 |
| **Fujian** |  |  |  |  |  |  |  |  |  |  |
| 10 to 14 | 0.01 | 0 | 0 | 0.01 | 0 | 0.01 | 0 | 0.01 | 0 | 0.04 |
| 15 to 19 | 0.08 | 0.03 | 0.02 | 0.11 | 0 | 0.13 | 0.08 | 0.04 | 0.02 | 0.27 |
| 20 to 24 | 0.32 | 0.13 | 0.04 | 0.21 | 0 | 0.64 | 0.35 | 0.16 | 0.05 | 1.09 |
| 25 to 29 | 1.15 | 0.47 | 0.12 | 0.7 | 0 | 2.56 | 1.31 | 0.58 | 0.17 | 4.18 |
| 30 to 34 | 1.11 | 0.41 | 0.12 | 0.75 | 0 | 3.03 | 1.21 | 0.72 | 0.17 | 4.36 |
| 35 to 39 | 0.63 | 0.2 | 0.07 | 0.45 | 0 | 1.66 | 0.59 | 0.39 | 0.12 | 2.27 |
| 40 to 44 | 0.33 | 0.13 | 0.04 | 0.37 | 0 | 0.73 | 0.28 | 0.2 | 0.13 | 1.05 |
| 45 to 49 | 0.11 | 0.03 | 0.03 | 0.38 | 0 | 0.17 | 0.1 | 0.15 | 0.19 | 0.62 |
| 50 to 54 | 0.03 | 0.01 | 0.01 | 0.07 | 0 | 0.03 | 0.02 | 0.03 | 0.05 | 0.17 |
| **Gansu** |  |  |  |  |  |  |  |  |  |  |
| 10 to 14 | 0.02 | 0.01 | 0.01 | 0.03 | 0 | 0.04 | 0.02 | 0.03 | 0.01 | 0.1 |
| 15 to 19 | 0.16 | 0.12 | 0.03 | 0.28 | 0 | 0.45 | 0.38 | 0.12 | 0.12 | 0.6 |
| 20 to 24 | 0.91 | 0.75 | 0.09 | 0.74 | 0 | 2.76 | 2.05 | 0.56 | 0.47 | 3.27 |
| 25 to 29 | 2.6 | 2.03 | 0.23 | 1.93 | 0 | 8.58 | 5.6 | 1.73 | 1.16 | 9.24 |
| 30 to 34 | 1.79 | 1.27 | 0.17 | 1.49 | 0 | 7.52 | 3.66 | 1.38 | 0.81 | 6.58 |
| 35 to 39 | 0.67 | 0.45 | 0.06 | 0.58 | 0 | 2.53 | 1.19 | 0.52 | 0.35 | 2.31 |
| 40 to 44 | 0.36 | 0.27 | 0.04 | 0.49 | 0 | 1.13 | 0.55 | 0.27 | 0.3 | 1.07 |
| 45 to 49 | 0.19 | 0.16 | 0.04 | 0.79 | 0 | 0.37 | 0.31 | 0.32 | 0.6 | 0.96 |
| 50 to 54 | 0.1 | 0.09 | 0.01 | 0.16 | 0 | 0.1 | 0.07 | 0.08 | 0.29 | 0.28 |
| **Guangdong** |  |  |  |  |  |  |  |  |  |  |
| 10 to 14 | 0.01 | 0.02 | 0.01 | 0.04 | 0 | 0.04 | 0.02 | 0.02 | 0.01 | 0.14 |
| 15 to 19 | 0.31 | 0.38 | 0.11 | 0.52 | 0 | 0.88 | 0.6 | 0.14 | 0.15 | 1.52 |
| 20 to 24 | 1.25 | 1.82 | 0.27 | 1.02 | 0 | 3.78 | 2.45 | 0.57 | 0.43 | 6.43 |
| 25 to 29 | 2.96 | 4.33 | 0.57 | 2.17 | 0 | 11.07 | 6.32 | 1.53 | 0.98 | 16.27 |
| 30 to 34 | 2.96 | 3.52 | 0.56 | 2.4 | 0 | 13.42 | 5.75 | 1.63 | 0.96 | 16.62 |
| 35 to 39 | 1.66 | 1.63 | 0.35 | 1.6 | 0 | 7.24 | 2.96 | 0.99 | 0.63 | 9.12 |
| 40 to 44 | 0.83 | 0.76 | 0.18 | 1.2 | 0 | 2.89 | 1.28 | 0.48 | 0.54 | 3.7 |
| 45 to 49 | 0.32 | 0.18 | 0.13 | 1.57 | 0 | 0.87 | 0.52 | 0.41 | 0.8 | 2.44 |
| 50 to 54 | 0.14 | 0.06 | 0.04 | 0.31 | 0 | 0.2 | 0.1 | 0.1 | 0.34 | 0.71 |
| **Guangxi** |  |  |  |  |  |  |  |  |  |  |
| 10 to 14 | 0.02 | 0.04 | 0.01 | 0.07 | 0 | 0.05 | 0.02 | 0.02 | 0.01 | 0.22 |
| 15 to 19 | 0.14 | 0.36 | 0.05 | 0.47 | 0 | 0.45 | 0.37 | 0.09 | 0.12 | 1.05 |
| 20 to 24 | 0.43 | 1.43 | 0.08 | 0.72 | 0 | 1.6 | 1.15 | 0.28 | 0.27 | 3.11 |
| 25 to 29 | 1.18 | 3.55 | 0.2 | 1.87 | 0.01 | 4.89 | 3.21 | 0.9 | 0.65 | 8.83 |
| 30 to 34 | 1.16 | 2.95 | 0.19 | 1.89 | 0.02 | 5.67 | 3.1 | 0.81 | 0.68 | 9.16 |
| 35 to 39 | 0.78 | 1.77 | 0.13 | 1.33 | 0.01 | 3.55 | 1.78 | 0.58 | 0.53 | 5.84 |
| 40 to 44 | 0.31 | 0.66 | 0.06 | 0.95 | 0 | 1.21 | 0.75 | 0.23 | 0.41 | 2.31 |
| 45 to 49 | 0.09 | 0.22 | 0.04 | 1.2 | 0 | 0.38 | 0.19 | 0.15 | 0.36 | 0.95 |
| 50 to 54 | 0.02 | 0.03 | 0.01 | 0.25 | 0 | 0.09 | 0.04 | 0.03 | 0.25 | 0.46 |
| **Guizhou** |  |  |  |  |  |  |  |  |  |  |
| 10 to 14 | 0.01 | 0.02 | 0 | 0.06 | 0 | 0.05 | 0.02 | 0.04 | 0.01 | 0.12 |
| 15 to 19 | 0.26 | 0.49 | 0.06 | 1.2 | 0 | 1.2 | 0.74 | 0.43 | 0.41 | 1.61 |
| 20 to 24 | 0.79 | 1.63 | 0.1 | 1.77 | 0 | 3.57 | 2.3 | 1.04 | 0.83 | 4.62 |
| 25 to 29 | 1.07 | 2.13 | 0.14 | 2.51 | 0.01 | 5.73 | 3.26 | 1.63 | 1.06 | 6.69 |
| 30 to 34 | 0.93 | 1.59 | 0.13 | 2.38 | 0.01 | 6.07 | 2.76 | 1.71 | 0.93 | 6.11 |
| 35 to 39 | 0.72 | 1.06 | 0.1 | 1.93 | 0.01 | 4.43 | 1.89 | 1.36 | 0.88 | 4.64 |
| 40 to 44 | 0.35 | 0.52 | 0.06 | 1.61 | 0 | 1.79 | 1.04 | 0.81 | 0.93 | 2.36 |
| 45 to 49 | 0.07 | 0.15 | 0.03 | 1.65 | 0 | 0.43 | 0.19 | 0.33 | 0.61 | 0.69 |
| 50 to 54 | 0.02 | 0.02 | 0.01 | 0.34 | 0 | 0.15 | 0.04 | 0.1 | 0.49 | 0.27 |
| **Hainan** |  |  |  |  |  |  |  |  |  |  |
| 10 to 14 | 0 | 0 | 0 | 0.01 | 0 | 0.01 | 0 | 0 | 0 | 0.02 |
| 15 to 19 | 0.04 | 0.07 | 0.01 | 0.17 | 0 | 0.11 | 0.15 | 0.03 | 0.04 | 0.16 |
| 20 to 24 | 0.16 | 0.36 | 0.03 | 0.37 | 0 | 0.52 | 0.6 | 0.13 | 0.13 | 0.66 |
| 25 to 29 | 0.31 | 0.63 | 0.04 | 0.62 | 0 | 1.13 | 1.12 | 0.3 | 0.22 | 1.27 |
| 30 to 34 | 0.3 | 0.54 | 0.04 | 0.66 | 0 | 1.33 | 1.06 | 0.28 | 0.23 | 1.33 |
| 35 to 39 | 0.18 | 0.28 | 0.03 | 0.46 | 0 | 0.78 | 0.57 | 0.19 | 0.16 | 0.77 |
| 40 to 44 | 0.07 | 0.13 | 0.01 | 0.32 | 0 | 0.25 | 0.21 | 0.08 | 0.11 | 0.27 |
| 45 to 49 | 0.02 | 0.02 | 0.01 | 0.26 | 0 | 0.04 | 0.05 | 0.04 | 0.1 | 0.1 |
| 50 to 54 | 0.01 | 0.01 | 0 | 0.05 | 0 | 0.01 | 0.01 | 0.01 | 0.05 | 0.05 |
| **Hebei** |  |  |  |  |  |  |  |  |  |  |
| 10 to 14 | 0.02 | 0.02 | 0.02 | 0.07 | 0 | 0.06 | 0.03 | 0.03 | 0.01 | 0.19 |
| 15 to 19 | 0.2 | 0.19 | 0.08 | 0.55 | 0 | 0.63 | 0.6 | 0.12 | 0.17 | 1.25 |
| 20 to 24 | 0.87 | 1 | 0.18 | 1.05 | 0 | 2.89 | 2.63 | 0.5 | 0.52 | 5.03 |
| 25 to 29 | 1.97 | 2.29 | 0.36 | 2.26 | 0 | 7.66 | 5.97 | 1.12 | 1.18 | 12.27 |
| 30 to 34 | 2 | 1.98 | 0.37 | 2.63 | 0 | 9.79 | 5.81 | 1.28 | 1.28 | 12.15 |
| 35 to 39 | 1.18 | 1.05 | 0.24 | 1.77 | 0 | 5.59 | 3.1 | 0.84 | 0.94 | 7.2 |
| 40 to 44 | 0.59 | 0.51 | 0.13 | 1.38 | 0 | 2.23 | 1.33 | 0.39 | 0.81 | 3.18 |
| 45 to 49 | 0.17 | 0.09 | 0.09 | 1.53 | 0 | 0.54 | 0.43 | 0.27 | 1.04 | 1.53 |
| 50 to 54 | 0.09 | 0.05 | 0.03 | 0.33 | 0 | 0.16 | 0.1 | 0.08 | 0.53 | 0.55 |
| **Heilongjiang** |  |  |  |  |  |  |  |  |  |  |
| 10 to 14 | 0.01 | 0.01 | 0 | 0.01 | 0 | 0.01 | 0.01 | 0.01 | 0 | 0.04 |
| 15 to 19 | 0.04 | 0.03 | 0.01 | 0.05 | 0 | 0.07 | 0.07 | 0.03 | 0.04 | 0.16 |
| 20 to 24 | 0.19 | 0.15 | 0.02 | 0.13 | 0 | 0.35 | 0.32 | 0.12 | 0.13 | 0.7 |
| 25 to 29 | 0.53 | 0.44 | 0.06 | 0.4 | 0 | 1.33 | 0.96 | 0.44 | 0.37 | 2.23 |
| 30 to 34 | 0.64 | 0.41 | 0.07 | 0.48 | 0 | 1.76 | 1.1 | 0.56 | 0.45 | 2.66 |
| 35 to 39 | 0.32 | 0.18 | 0.04 | 0.26 | 0 | 0.81 | 0.51 | 0.26 | 0.26 | 1.36 |
| 40 to 44 | 0.19 | 0.1 | 0.03 | 0.28 | 0 | 0.43 | 0.31 | 0.2 | 0.33 | 0.79 |
| 45 to 49 | 0.05 | 0.02 | 0.02 | 0.33 | 0 | 0.12 | 0.06 | 0.09 | 0.24 | 0.6 |
| 50 to 54 | 0.01 | 0 | 0.01 | 0.04 | 0 | 0.01 | 0.01 | 0.01 | 0.12 | 0.27 |
| **Henan** |  |  |  |  |  |  |  |  |  |  |
| 10 to 14 | 0.04 | 0.04 | 0.03 | 0.11 | 0 | 0.14 | 0.07 | 0.11 | 0.01 | 0.29 |
| 15 to 19 | 0.23 | 0.19 | 0.07 | 0.5 | 0 | 0.89 | 0.74 | 0.3 | 0.11 | 0.98 |
| 20 to 24 | 0.96 | 0.84 | 0.17 | 0.94 | 0 | 3.67 | 2.98 | 1.01 | 0.3 | 3.71 |
| 25 to 29 | 4.55 | 4.02 | 0.72 | 4.47 | 0.01 | 22.15 | 14.38 | 5.69 | 1.3 | 18.69 |
| 30 to 34 | 3.15 | 2.29 | 0.48 | 2.93 | 0.01 | 17.12 | 8.99 | 4.59 | 0.82 | 12.9 |
| 35 to 39 | 1.57 | 0.92 | 0.26 | 1.75 | 0 | 7.97 | 4.09 | 2.36 | 0.49 | 6.61 |
| 40 to 44 | 0.75 | 0.35 | 0.15 | 1.6 | 0 | 3.42 | 2.09 | 1.42 | 0.44 | 3.09 |
| 45 to 49 | 0.42 | 0.03 | 0.15 | 3.81 | 0 | 1.72 | 0.71 | 1.21 | 0.53 | 2.27 |
| 50 to 54 | 0.03 | 0.06 | 0.06 | 0.61 | 0 | 0.36 | 0.16 | 0.27 | 0.97 | 1.31 |
| **Hong Kong** |  |  |  |  |  |  |  |  |  |  |
| 10 to 14 | 0 | 0 | 0 | 0 | 0 | 0 | 0 | 0 | 0 | 0 |
| 15 to 19 | 0.01 | 0 | 0 | 0.02 | 0 | 0.03 | 0.01 | 0 | 0.01 | 0.03 |
| 20 to 24 | 0.05 | 0.02 | 0.01 | 0.06 | 0 | 0.25 | 0.09 | 0.03 | 0.04 | 0.2 |
| 25 to 29 | 0.07 | 0.03 | 0.02 | 0.09 | 0 | 0.39 | 0.15 | 0.05 | 0.05 | 0.35 |
| 30 to 34 | 0.09 | 0.03 | 0.02 | 0.1 | 0 | 0.49 | 0.18 | 0.07 | 0.08 | 0.48 |
| 35 to 39 | 0.09 | 0.03 | 0.03 | 0.11 | 0 | 0.41 | 0.17 | 0.07 | 0.1 | 0.44 |
| 40 to 44 | 0.04 | 0.01 | 0.02 | 0.1 | 0 | 0.19 | 0.07 | 0.03 | 0.1 | 0.19 |
| 45 to 49 | 0.01 | 0 | 0.01 | 0.1 | 0 | 0.05 | 0.01 | 0.01 | 0.08 | 0.08 |
| 50 to 54 | 0 | 0 | 0 | 0.02 | 0 | 0.01 | 0 | 0.01 | 0.03 | 0.04 |
| **Hubei** |  |  |  |  |  |  |  |  |  |  |
| 10 to 14 | 0.01 | 0.01 | 0 | 0.02 | 0 | 0.01 | 0.01 | 0.01 | 0 | 0.04 |
| 15 to 19 | 0.05 | 0.04 | 0.01 | 0.1 | 0 | 0.07 | 0.07 | 0.04 | 0.03 | 0.12 |
| 20 to 24 | 0.43 | 0.46 | 0.05 | 0.4 | 0 | 0.61 | 0.62 | 0.24 | 0.18 | 0.96 |
| 25 to 29 | 1.81 | 1.87 | 0.17 | 1.68 | 0 | 3.09 | 2.52 | 1.42 | 0.73 | 4.17 |
| 30 to 34 | 1.56 | 1.41 | 0.15 | 1.62 | 0 | 3.13 | 2.09 | 1.47 | 0.66 | 3.76 |
| 35 to 39 | 0.74 | 0.54 | 0.08 | 0.82 | 0 | 1.34 | 0.9 | 0.65 | 0.39 | 1.84 |
| 40 to 44 | 0.31 | 0.2 | 0.04 | 0.7 | 0 | 0.52 | 0.4 | 0.33 | 0.33 | 0.82 |
| 45 to 49 | 0.11 | 0.06 | 0.04 | 1.06 | 0 | 0.24 | 0.11 | 0.23 | 0.3 | 0.54 |
| 50 to 54 | 0.01 | 0.02 | 0.02 | 0.16 | 0 | 0.05 | 0.03 | 0.04 | 0.18 | 0.51 |
| **Hunan** |  |  |  |  |  |  |  |  |  |  |
| 10 to 14 | 0.03 | 0.03 | 0.02 | 0.06 | 0 | 0.07 | 0.04 | 0.05 | 0.03 | 0.24 |
| 15 to 19 | 0.18 | 0.22 | 0.05 | 0.27 | 0 | 0.57 | 0.46 | 0.15 | 0.27 | 1.04 |
| 20 to 24 | 0.43 | 0.67 | 0.08 | 0.31 | 0 | 1.65 | 1.16 | 0.32 | 0.44 | 2.55 |
| 25 to 29 | 1.43 | 2.17 | 0.24 | 1 | 0 | 6.64 | 4.05 | 1.37 | 1.39 | 8.94 |
| 30 to 34 | 1.64 | 2.09 | 0.28 | 1.23 | 0.01 | 9.45 | 4.38 | 1.62 | 1.67 | 10.94 |
| 35 to 39 | 0.77 | 0.84 | 0.14 | 0.66 | 0 | 4.03 | 1.88 | 0.8 | 0.94 | 5.03 |
| 40 to 44 | 0.42 | 0.42 | 0.09 | 0.62 | 0 | 1.74 | 1.04 | 0.49 | 0.96 | 2.58 |
| 45 to 49 | 0.17 | 0.11 | 0.06 | 0.98 | 0 | 0.59 | 0.32 | 0.35 | 1.22 | 1.41 |
| 50 to 54 | 0.03 | 0.03 | 0.03 | 0.23 | 0 | 0.14 | 0.06 | 0.1 | 0.9 | 0.62 |
| **Inner Mongolia** |  |  |  |  |  |  |  |  |  |  |
| 10 to 14 | 0 | 0 | 0 | 0.01 | 0 | 0.01 | 0.01 | 0 | 0 | 0.04 |
| 15 to 19 | 0.04 | 0.02 | 0.01 | 0.05 | 0 | 0.07 | 0.09 | 0.01 | 0.02 | 0.19 |
| 20 to 24 | 0.18 | 0.09 | 0.03 | 0.11 | 0 | 0.34 | 0.41 | 0.05 | 0.07 | 0.81 |
| 25 to 29 | 0.56 | 0.3 | 0.07 | 0.34 | 0 | 1.26 | 1.36 | 0.19 | 0.21 | 2.72 |
| 30 to 34 | 0.62 | 0.27 | 0.08 | 0.38 | 0 | 1.58 | 1.34 | 0.22 | 0.23 | 3.08 |
| 35 to 39 | 0.29 | 0.11 | 0.04 | 0.2 | 0 | 0.72 | 0.55 | 0.11 | 0.13 | 1.41 |
| 40 to 44 | 0.21 | 0.1 | 0.03 | 0.25 | 0 | 0.46 | 0.38 | 0.08 | 0.2 | 0.88 |
| 45 to 49 | 0.06 | 0.02 | 0.02 | 0.23 | 0 | 0.09 | 0.12 | 0.05 | 0.26 | 0.42 |
| 50 to 54 | 0.03 | 0.01 | 0.01 | 0.05 | 0 | 0.02 | 0.02 | 0.01 | 0.08 | 0.14 |
| **Jiangsu** |  |  |  |  |  |  |  |  |  |  |
| 10 to 14 | 0.01 | 0.01 | 0.01 | 0.02 | 0 | 0.02 | 0.01 | 0.01 | 0 | 0.06 |
| 15 to 19 | 0.04 | 0.02 | 0.02 | 0.1 | 0 | 0.13 | 0.07 | 0.02 | 0.03 | 0.23 |
| 20 to 24 | 0.22 | 0.16 | 0.05 | 0.25 | 0 | 0.81 | 0.36 | 0.1 | 0.1 | 1.25 |
| 25 to 29 | 0.92 | 0.64 | 0.21 | 1.09 | 0 | 4.35 | 1.58 | 0.49 | 0.4 | 5.6 |
| 30 to 34 | 0.62 | 0.36 | 0.14 | 0.83 | 0 | 3.62 | 1.02 | 0.36 | 0.28 | 3.87 |
| 35 to 39 | 0.46 | 0.22 | 0.11 | 0.64 | 0 | 2.29 | 0.65 | 0.28 | 0.26 | 2.76 |
| 40 to 44 | 0.15 | 0.07 | 0.05 | 0.4 | 0 | 0.73 | 0.23 | 0.11 | 0.2 | 0.94 |
| 45 to 49 | 0.07 | 0.02 | 0.06 | 0.83 | 0 | 0.35 | 0.08 | 0.08 | 0.25 | 1.14 |
| 50 to 54 | 0.01 | 0.01 | 0.02 | 0.11 | 0 | 0.04 | 0.01 | 0.02 | 0.14 | 0.61 |
| **Jiangxi** |  |  |  |  |  |  |  |  |  |  |
| 10 to 14 | 0.01 | 0.02 | 0.01 | 0.02 | 0 | 0.03 | 0.02 | 0.02 | 0 | 0.1 |
| 15 to 19 | 0.11 | 0.18 | 0.03 | 0.21 | 0 | 0.37 | 0.35 | 0.08 | 0.06 | 0.66 |
| 20 to 24 | 0.39 | 0.8 | 0.07 | 0.35 | 0 | 1.47 | 1.28 | 0.29 | 0.16 | 2.32 |
| 25 to 29 | 1.07 | 2.21 | 0.16 | 0.88 | 0 | 4.78 | 3.51 | 0.78 | 0.43 | 6.7 |
| 30 to 34 | 0.9 | 1.42 | 0.14 | 0.78 | 0 | 4.82 | 2.67 | 0.73 | 0.36 | 5.81 |
| 35 to 39 | 0.5 | 0.69 | 0.08 | 0.49 | 0 | 2.56 | 1.33 | 0.41 | 0.26 | 2.97 |
| 40 to 44 | 0.38 | 0.54 | 0.06 | 0.56 | 0 | 1.68 | 0.83 | 0.28 | 0.34 | 1.79 |
| 45 to 49 | 0.1 | 0.07 | 0.03 | 0.46 | 0 | 0.27 | 0.23 | 0.17 | 0.42 | 0.83 |
| 50 to 54 | 0.06 | 0.03 | 0.01 | 0.09 | 0 | 0.06 | 0.05 | 0.05 | 0.15 | 0.27 |
| **Jilin** |  |  |  |  |  |  |  |  |  |  |
| 10 to 14 | 0 | 0 | 0 | 0.01 | 0 | 0.01 | 0.01 | 0 | 0 | 0.03 |
| 15 to 19 | 0.04 | 0.04 | 0.02 | 0.11 | 0 | 0.12 | 0.14 | 0.02 | 0.06 | 0.22 |
| 20 to 24 | 0.19 | 0.24 | 0.04 | 0.25 | 0 | 0.68 | 0.67 | 0.1 | 0.21 | 1.11 |
| 25 to 29 | 0.44 | 0.56 | 0.09 | 0.52 | 0 | 1.81 | 1.55 | 0.32 | 0.45 | 2.75 |
| 30 to 34 | 0.45 | 0.47 | 0.09 | 0.59 | 0 | 2.21 | 1.5 | 0.3 | 0.46 | 2.94 |
| 35 to 39 | 0.3 | 0.27 | 0.06 | 0.45 | 0 | 1.42 | 0.87 | 0.2 | 0.36 | 1.71 |
| 40 to 44 | 0.16 | 0.15 | 0.04 | 0.36 | 0 | 0.58 | 0.4 | 0.1 | 0.32 | 0.77 |
| 45 to 49 | 0.05 | 0.03 | 0.02 | 0.35 | 0 | 0.13 | 0.12 | 0.07 | 0.35 | 0.41 |
| 50 to 54 | 0.01 | 0.01 | 0.01 | 0.06 | 0 | 0.02 | 0.02 | 0.01 | 0.16 | 0.14 |
| **Liaoning** |  |  |  |  |  |  |  |  |  |  |
| 10 to 14 | 0 | 0.01 | 0 | 0.02 | 0 | 0.01 | 0.01 | 0 | 0 | 0.05 |
| 15 to 19 | 0.03 | 0.04 | 0.01 | 0.11 | 0 | 0.06 | 0.08 | 0.01 | 0.04 | 0.22 |
| 20 to 24 | 0.13 | 0.2 | 0.03 | 0.21 | 0 | 0.28 | 0.31 | 0.03 | 0.11 | 0.82 |
| 25 to 29 | 0.55 | 0.81 | 0.11 | 0.85 | 0 | 1.45 | 1.29 | 0.12 | 0.45 | 3.6 |
| 30 to 34 | 0.49 | 0.59 | 0.1 | 0.74 | 0 | 1.47 | 1.11 | 0.12 | 0.43 | 3.42 |
| 35 to 39 | 0.31 | 0.34 | 0.07 | 0.52 | 0 | 0.91 | 0.66 | 0.08 | 0.36 | 2.25 |
| 40 to 44 | 0.14 | 0.15 | 0.04 | 0.42 | 0 | 0.34 | 0.34 | 0.05 | 0.38 | 1.05 |
| 45 to 49 | 0.03 | 0.02 | 0.03 | 0.56 | 0 | 0.1 | 0.07 | 0.02 | 0.29 | 0.53 |
| 50 to 54 | 0 | 0.01 | 0.01 | 0.09 | 0 | 0.02 | 0.01 | 0 | 0.1 | 0.31 |
| **Macao** |  |  |  |  |  |  |  |  |  |  |
| 10 to 14 | 0 | 0 | 0 | 0 | 0 | 0 | 0 | 0 | 0 | 0 |
| 15 to 19 | 0 | 0 | 0 | 0 | 0 | 0 | 0 | 0 | 0 | 0 |
| 20 to 24 | 0.01 | 0 | 0 | 0.02 | 0 | 0.03 | 0.02 | 0 | 0.01 | 0.04 |
| 25 to 29 | 0.01 | 0 | 0 | 0.02 | 0 | 0.04 | 0.03 | 0.01 | 0.01 | 0.07 |
| 30 to 34 | 0.01 | 0 | 0 | 0.02 | 0 | 0.04 | 0.02 | 0.01 | 0.01 | 0.07 |
| 35 to 39 | 0 | 0 | 0 | 0.02 | 0 | 0.03 | 0.02 | 0 | 0.01 | 0.05 |
| 40 to 44 | 0 | 0 | 0 | 0.01 | 0 | 0.01 | 0 | 0 | 0.01 | 0.01 |
| 45 to 49 | 0 | 0 | 0 | 0.01 | 0 | 0 | 0 | 0 | 0.01 | 0.01 |
| 50 to 54 | 0 | 0 | 0 | 0 | 0 | 0 | 0 | 0 | 0 | 0 |
| **Ningxia** |  |  |  |  |  |  |  |  |  |  |
| 10 to 14 | 0 | 0 | 0 | 0 | 0 | 0 | 0 | 0 | 0 | 0.01 |
| 15 to 19 | 0.01 | 0.07 | 0.01 | 0.06 | 0 | 0.07 | 0.07 | 0.02 | 0.03 | 0.12 |
| 20 to 24 | 0.06 | 0.31 | 0.02 | 0.13 | 0 | 0.31 | 0.29 | 0.07 | 0.09 | 0.48 |
| 25 to 29 | 0.11 | 0.51 | 0.03 | 0.21 | 0 | 0.64 | 0.5 | 0.14 | 0.14 | 0.87 |
| 30 to 34 | 0.11 | 0.42 | 0.03 | 0.23 | 0 | 0.8 | 0.5 | 0.14 | 0.14 | 0.88 |
| 35 to 39 | 0.07 | 0.27 | 0.02 | 0.17 | 0 | 0.53 | 0.31 | 0.09 | 0.11 | 0.59 |
| 40 to 44 | 0.03 | 0.17 | 0.01 | 0.11 | 0 | 0.24 | 0.12 | 0.04 | 0.09 | 0.23 |
| 45 to 49 | 0.01 | 0.02 | 0.01 | 0.11 | 0 | 0.04 | 0.04 | 0.03 | 0.1 | 0.12 |
| 50 to 54 | 0 | 0.01 | 0 | 0.02 | 0 | 0.01 | 0.01 | 0.01 | 0.04 | 0.03 |
| **Qinghai** |  |  |  |  |  |  |  |  |  |  |
| 10 to 14 | 0 | 0 | 0 | 0.01 | 0 | 0.01 | 0.01 | 0 | 0 | 0.03 |
| 15 to 19 | 0.05 | 0.09 | 0.01 | 0.15 | 0 | 0.33 | 0.42 | 0.04 | 0.08 | 0.3 |
| 20 to 24 | 0.2 | 0.37 | 0.03 | 0.26 | 0 | 1.34 | 1.52 | 0.12 | 0.19 | 0.98 |
| 25 to 29 | 0.35 | 0.62 | 0.06 | 0.45 | 0 | 2.73 | 2.77 | 0.36 | 0.32 | 1.88 |
| 30 to 34 | 0.31 | 0.46 | 0.05 | 0.42 | 0 | 2.89 | 2.22 | 0.22 | 0.27 | 1.73 |
| 35 to 39 | 0.23 | 0.29 | 0.04 | 0.36 | 0 | 2.17 | 1.46 | 0.17 | 0.24 | 1.18 |
| 40 to 44 | 0.14 | 0.18 | 0.02 | 0.3 | 0 | 1.07 | 0.74 | 0.09 | 0.21 | 0.54 |
| 45 to 49 | 0.05 | 0.03 | 0.01 | 0.3 | 0 | 0.19 | 0.25 | 0.07 | 0.29 | 0.31 |
| 50 to 54 | 0.02 | 0.01 | 0 | 0.06 | 0 | 0.04 | 0.03 | 0.01 | 0.15 | 0.09 |
| **Shaanxi** |  |  |  |  |  |  |  |  |  |  |
| 10 to 14 | 0.01 | 0.01 | 0 | 0.01 | 0 | 0.02 | 0.01 | 0.01 | 0 | 0.06 |
| 15 to 19 | 0.15 | 0.14 | 0.04 | 0.19 | 0 | 0.49 | 0.34 | 0.13 | 0.1 | 0.7 |
| 20 to 24 | 0.79 | 0.91 | 0.11 | 0.46 | 0 | 2.84 | 1.86 | 0.65 | 0.37 | 3.72 |
| 25 to 29 | 1.7 | 1.85 | 0.23 | 0.89 | 0 | 7.15 | 4.06 | 2.05 | 0.73 | 8.53 |
| 30 to 34 | 1.67 | 1.62 | 0.22 | 0.99 | 0 | 8.36 | 3.81 | 1.55 | 0.73 | 8.91 |
| 35 to 39 | 0.88 | 0.67 | 0.12 | 0.6 | 0 | 4.36 | 1.77 | 0.87 | 0.45 | 4.07 |
| 40 to 44 | 0.39 | 0.31 | 0.07 | 0.46 | 0 | 2.05 | 0.76 | 0.43 | 0.38 | 1.84 |
| 45 to 49 | 0.16 | 0.07 | 0.04 | 0.54 | 0 | 0.49 | 0.29 | 0.36 | 0.53 | 1.14 |
| 50 to 54 | 0.05 | 0.02 | 0.01 | 0.1 | 0 | 0.08 | 0.05 | 0.07 | 0.28 | 0.32 |
| **Shandong** |  |  |  |  |  |  |  |  |  |  |
| 10 to 14 | 0.03 | 0.01 | 0.02 | 0.06 | 0 | 0.05 | 0.02 | 0.03 | 0.01 | 0.16 |
| 15 to 19 | 0.2 | 0.08 | 0.06 | 0.37 | 0 | 0.44 | 0.31 | 0.1 | 0.1 | 0.78 |
| 20 to 24 | 0.51 | 0.26 | 0.08 | 0.42 | 0 | 1.22 | 0.81 | 0.23 | 0.18 | 1.97 |
| 25 to 29 | 2.77 | 1.34 | 0.39 | 2.14 | 0 | 7.66 | 4.31 | 1.57 | 0.98 | 11.1 |
| 30 to 34 | 2.79 | 1.14 | 0.4 | 2.35 | 0 | 9.29 | 4.18 | 1.63 | 1.03 | 12.11 |
| 35 to 39 | 1.38 | 0.47 | 0.22 | 1.23 | 0 | 4.26 | 1.93 | 0.88 | 0.65 | 5.86 |
| 40 to 44 | 0.6 | 0.18 | 0.12 | 0.98 | 0 | 1.44 | 0.9 | 0.46 | 0.63 | 2.7 |
| 45 to 49 | 0.19 | 0.07 | 0.11 | 1.96 | 0 | 0.64 | 0.28 | 0.3 | 0.78 | 1.94 |
| 50 to 54 | 0.03 | 0.01 | 0.04 | 0.28 | 0 | 0.09 | 0.05 | 0.07 | 0.34 | 1.04 |
| **Shanghai** |  |  |  |  |  |  |  |  |  |  |
| 10 to 14 | 0 | 0.01 | 0 | 0 | 0 | 0 | 0 | 0 | 0 | 0.02 |
| 15 to 19 | 0.01 | 0.08 | 0.01 | 0.02 | 0 | 0.03 | 0.03 | 0.02 | 0.01 | 0.13 |
| 20 to 24 | 0.04 | 0.33 | 0.01 | 0.02 | 0 | 0.11 | 0.1 | 0.06 | 0.03 | 0.48 |
| 25 to 29 | 0.1 | 0.84 | 0.03 | 0.07 | 0 | 0.34 | 0.28 | 0.18 | 0.07 | 1.39 |
| 30 to 34 | 0.15 | 1.03 | 0.04 | 0.1 | 0 | 0.6 | 0.39 | 0.26 | 0.11 | 2.11 |
| 35 to 39 | 0.08 | 0.46 | 0.02 | 0.07 | 0 | 0.3 | 0.18 | 0.12 | 0.07 | 1.01 |
| 40 to 44 | 0.04 | 0.2 | 0.01 | 0.05 | 0 | 0.12 | 0.08 | 0.06 | 0.07 | 0.42 |
| 45 to 49 | 0.02 | 0.04 | 0.01 | 0.05 | 0 | 0.03 | 0.03 | 0.05 | 0.08 | 0.23 |
| 50 to 54 | 0.01 | 0.02 | 0 | 0.01 | 0 | 0.01 | 0.01 | 0.01 | 0.02 | 0.07 |
| **Shanxi** |  |  |  |  |  |  |  |  |  |  |
| 10 to 14 | 0.01 | 0.01 | 0 | 0.01 | 0 | 0.02 | 0.01 | 0.01 | 0 | 0.06 |
| 15 to 19 | 0.17 | 0.18 | 0.05 | 0.16 | 0 | 0.46 | 0.39 | 0.08 | 0.17 | 0.76 |
| 20 to 24 | 0.88 | 0.99 | 0.14 | 0.37 | 0 | 2.57 | 2 | 0.34 | 0.6 | 3.82 |
| 25 to 29 | 1.53 | 1.6 | 0.21 | 0.58 | 0 | 5.18 | 3.5 | 0.84 | 1.02 | 7.05 |
| 30 to 34 | 1.55 | 1.41 | 0.21 | 0.62 | 0 | 5.94 | 3.32 | 0.84 | 1.06 | 7.29 |
| 35 to 39 | 0.84 | 0.63 | 0.12 | 0.38 | 0 | 3.23 | 1.63 | 0.46 | 0.71 | 3.74 |
| 40 to 44 | 0.43 | 0.4 | 0.08 | 0.34 | 0 | 1.6 | 0.79 | 0.28 | 0.73 | 1.74 |
| 45 to 49 | 0.15 | 0.08 | 0.05 | 0.36 | 0 | 0.35 | 0.25 | 0.18 | 0.92 | 0.93 |
| 50 to 54 | 0.06 | 0.02 | 0.01 | 0.07 | 0 | 0.07 | 0.04 | 0.03 | 0.35 | 0.24 |
| **Sichuan** |  |  |  |  |  |  |  |  |  |  |
| 10 to 14 | 0.01 | 0.02 | 0.01 | 0.03 | 0 | 0.04 | 0.01 | 0.03 | 0.01 | 0.09 |
| 15 to 19 | 0.27 | 0.53 | 0.07 | 0.51 | 0 | 1.11 | 0.55 | 0.3 | 0.23 | 1.25 |
| 20 to 24 | 1.15 | 2.4 | 0.17 | 0.97 | 0 | 4.66 | 2.27 | 1.28 | 0.62 | 5.03 |
| 25 to 29 | 2.04 | 4.39 | 0.29 | 1.64 | 0.01 | 10.1 | 4.17 | 2.19 | 1.07 | 9.8 |
| 30 to 34 | 1.58 | 2.95 | 0.23 | 1.37 | 0.01 | 9.52 | 3.1 | 1.8 | 0.85 | 8.05 |
| 35 to 39 | 1 | 1.46 | 0.15 | 0.93 | 0.01 | 5.98 | 1.72 | 1.15 | 0.61 | 4.72 |
| 40 to 44 | 0.74 | 1.35 | 0.13 | 1.05 | 0 | 4.38 | 1.11 | 0.92 | 0.74 | 2.96 |
| 45 to 49 | 0.28 | 0.27 | 0.08 | 1.44 | 0 | 1.03 | 0.47 | 0.75 | 1.15 | 2.07 |
| 50 to 54 | 0.11 | 0.05 | 0.03 | 0.31 | 0 | 0.15 | 0.08 | 0.13 | 0.65 | 0.53 |
| **Taiwan** |  |  |  |  |  |  |  |  |  |  |
| 10 to 14 | 0 | 0 | 0 | 0 | 0 | 0 | 0 | 0 | 0 | 0.02 |
| 15 to 19 | 0.01 | 0.01 | 0.03 | 0.12 | 0 | 0.04 | 0.06 | 0 | 0.06 | 0.16 |
| 20 to 24 | 0.02 | 0.04 | 0.05 | 0.25 | 0 | 0.15 | 0.13 | 0.01 | 0.1 | 0.48 |
| 25 to 29 | 0.06 | 0.12 | 0.13 | 0.54 | 0 | 0.5 | 0.33 | 0.01 | 0.26 | 1.51 |
| 30 to 34 | 0.1 | 0.2 | 0.24 | 1.03 | 0 | 1.02 | 0.46 | 0.03 | 0.54 | 3.04 |
| 35 to 39 | 0.09 | 0.13 | 0.25 | 1.48 | 0 | 0.77 | 0.35 | 0.03 | 0.76 | 2.72 |
| 40 to 44 | 0.03 | 0.04 | 0.17 | 1.71 | 0 | 0.28 | 0.11 | 0.01 | 0.69 | 1.02 |
| 45 to 49 | 0 | 0 | 0.19 | 2.21 | 0 | 0.01 | 0 | 0 | 1.43 | 0.15 |
| 50 to 54 | 0 | 0 | 0.01 | 0.04 | 0 | 0.01 | 0 | 0 | 0.02 | 0.1 |
| **Tianjin** |  |  |  |  |  |  |  |  |  |  |
| 10 to 14 | 0 | 0 | 0 | 0 | 0 | 0 | 0 | 0 | 0 | 0.01 |
| 15 to 19 | 0.02 | 0.02 | 0.01 | 0.03 | 0 | 0.02 | 0.02 | 0.01 | 0 | 0.08 |
| 20 to 24 | 0.13 | 0.13 | 0.03 | 0.08 | 0 | 0.19 | 0.18 | 0.04 | 0.02 | 0.57 |
| 25 to 29 | 0.37 | 0.37 | 0.07 | 0.23 | 0 | 0.65 | 0.54 | 0.19 | 0.07 | 1.76 |
| 30 to 34 | 0.41 | 0.33 | 0.07 | 0.25 | 0 | 0.78 | 0.55 | 0.2 | 0.08 | 1.99 |
| 35 to 39 | 0.2 | 0.14 | 0.04 | 0.15 | 0 | 0.38 | 0.24 | 0.08 | 0.05 | 0.92 |
| 40 to 44 | 0.07 | 0.05 | 0.02 | 0.1 | 0 | 0.14 | 0.09 | 0.04 | 0.04 | 0.33 |
| 45 to 49 | 0.02 | 0.01 | 0.01 | 0.1 | 0 | 0.03 | 0.02 | 0.02 | 0.04 | 0.15 |
| 50 to 54 | 0.01 | 0 | 0 | 0.02 | 0 | 0.01 | 0.01 | 0.01 | 0.02 | 0.06 |
| **Tibet** |  |  |  |  |  |  |  |  |  |  |
| 10 to 14 | 0.01 | 0.01 | 0 | 0.01 | 0 | 0.02 | 0.02 | 0.01 | 0.01 | 0.05 |
| 15 to 19 | 0.15 | 0.42 | 0.02 | 0.22 | 0 | 0.69 | 0.76 | 0.06 | 0.15 | 0.5 |
| 20 to 24 | 0.37 | 0.68 | 0.03 | 0.26 | 0 | 1.21 | 1.78 | 0.14 | 0.28 | 1.14 |
| 25 to 29 | 1.02 | 1.81 | 0.09 | 0.61 | 0 | 3.89 | 4.8 | 0.44 | 0.73 | 3.26 |
| 30 to 34 | 1.11 | 1.53 | 0.1 | 0.73 | 0 | 4.98 | 4.72 | 0.53 | 0.79 | 3.44 |
| 35 to 39 | 0.61 | 0.91 | 0.05 | 0.45 | 0 | 3.08 | 2.44 | 0.3 | 0.55 | 1.87 |
| 40 to 44 | 0.16 | 0.28 | 0.02 | 0.19 | 0 | 1.08 | 0.64 | 0.08 | 0.3 | 0.46 |
| 45 to 49 | 0.04 | 0.03 | 0 | 0.12 | 0 | 0.08 | 0.16 | 0.04 | 0.29 | 0.17 |
| 50 to 54 | 0.04 | 0.02 | 0 | 0.04 | 0 | 0.03 | 0.05 | 0.02 | 0.12 | 0.08 |
| **Xinjiang** |  |  |  |  |  |  |  |  |  |  |
| 10 to 14 | 0.02 | 0.02 | 0.01 | 0.05 | 0 | 0.08 | 0.08 | 0.08 | 0.02 | 0.18 |
| 15 to 19 | 0.31 | 0.45 | 0.09 | 0.67 | 0 | 1.6 | 2.26 | 0.57 | 0.47 | 1.44 |
| 20 to 24 | 1.15 | 1.52 | 0.18 | 1.13 | 0.01 | 6.19 | 7.31 | 1.94 | 1.11 | 4.75 |
| 25 to 29 | 2.19 | 2.83 | 0.32 | 1.84 | 0.02 | 12.95 | 14.18 | 2.97 | 1.99 | 9.39 |
| 30 to 34 | 2 | 2.06 | 0.29 | 1.75 | 0.02 | 13.94 | 12.21 | 3.25 | 1.77 | 8.64 |
| 35 to 39 | 0.91 | 0.86 | 0.13 | 0.91 | 0.01 | 6.09 | 4.95 | 1.56 | 0.97 | 3.75 |
| 40 to 44 | 0.53 | 0.63 | 0.09 | 0.99 | 0.01 | 3.63 | 2.92 | 1.03 | 1.07 | 2.07 |
| 45 to 49 | 0.19 | 0.12 | 0.05 | 1.22 | 0 | 0.82 | 0.92 | 0.79 | 1.31 | 1.14 |
| 50 to 54 | 0.07 | 0.04 | 0.02 | 0.17 | 0 | 0.16 | 0.13 | 0.17 | 0.71 | 0.41 |
| **Yunnan** |  |  |  |  |  |  |  |  |  |  |
| 10 to 14 | 0.03 | 0.02 | 0.01 | 0.11 | 0 | 0.07 | 0.02 | 0.06 | 0.02 | 0.17 |
| 15 to 19 | 0.43 | 0.32 | 0.07 | 1.22 | 0 | 1.36 | 0.73 | 0.44 | 0.36 | 1.52 |
| 20 to 24 | 1.18 | 0.91 | 0.11 | 1.59 | 0.01 | 3.95 | 1.93 | 1.2 | 0.65 | 3.92 |
| 25 to 29 | 2.86 | 2.23 | 0.25 | 3.41 | 0.02 | 10.86 | 4.72 | 2.82 | 1.42 | 9.88 |
| 30 to 34 | 2.14 | 1.47 | 0.19 | 2.87 | 0.02 | 9.57 | 3.35 | 2.22 | 1.07 | 7.61 |
| 35 to 39 | 1.23 | 0.82 | 0.11 | 1.78 | 0.01 | 5.32 | 1.67 | 1.18 | 0.7 | 4.19 |
| 40 to 44 | 0.66 | 0.67 | 0.08 | 1.61 | 0.01 | 3.25 | 0.84 | 0.69 | 0.68 | 2.13 |
| 45 to 49 | 0.25 | 0.14 | 0.04 | 1.52 | 0 | 0.52 | 0.28 | 0.49 | 0.87 | 1.17 |
| 50 to 54 | 0.14 | 0.08 | 0.02 | 0.36 | 0 | 0.13 | 0.07 | 0.12 | 0.5 | 0.35 |
| **Zhejiang** |  |  |  |  |  |  |  |  |  |  |
| 10 to 14 | 0.01 | 0 | 0 | 0.01 | 0 | 0.01 | 0.01 | 0 | 0 | 0.04 |
| 15 to 19 | 0.04 | 0.03 | 0.02 | 0.07 | 0 | 0.11 | 0.1 | 0.01 | 0.02 | 0.22 |
| 20 to 24 | 0.17 | 0.13 | 0.04 | 0.13 | 0 | 0.51 | 0.39 | 0.03 | 0.04 | 0.86 |
| 25 to 29 | 0.5 | 0.36 | 0.1 | 0.34 | 0 | 1.71 | 1.16 | 0.15 | 0.13 | 2.8 |
| 30 to 34 | 0.55 | 0.33 | 0.11 | 0.42 | 0 | 2.32 | 1.18 | 0.12 | 0.14 | 3.2 |
| 35 to 39 | 0.39 | 0.19 | 0.08 | 0.32 | 0 | 1.59 | 0.72 | 0.1 | 0.12 | 2.11 |
| 40 to 44 | 0.18 | 0.08 | 0.05 | 0.28 | 0 | 0.67 | 0.33 | 0.05 | 0.12 | 0.94 |
| 45 to 49 | 0.07 | 0.02 | 0.03 | 0.31 | 0 | 0.17 | 0.12 | 0.04 | 0.19 | 0.58 |
| 50 to 54 | 0.03 | 0.01 | 0.01 | 0.07 | 0 | 0.04 | 0.03 | 0.01 | 0.07 | 0.25 |

**Supplementary figure legends**

**Fig. S1** Observed maternal deaths, and expected maternal deaths probabilistically predicted based on the Socio-demographic Index for the 34 provinces, from 1990 to 2017


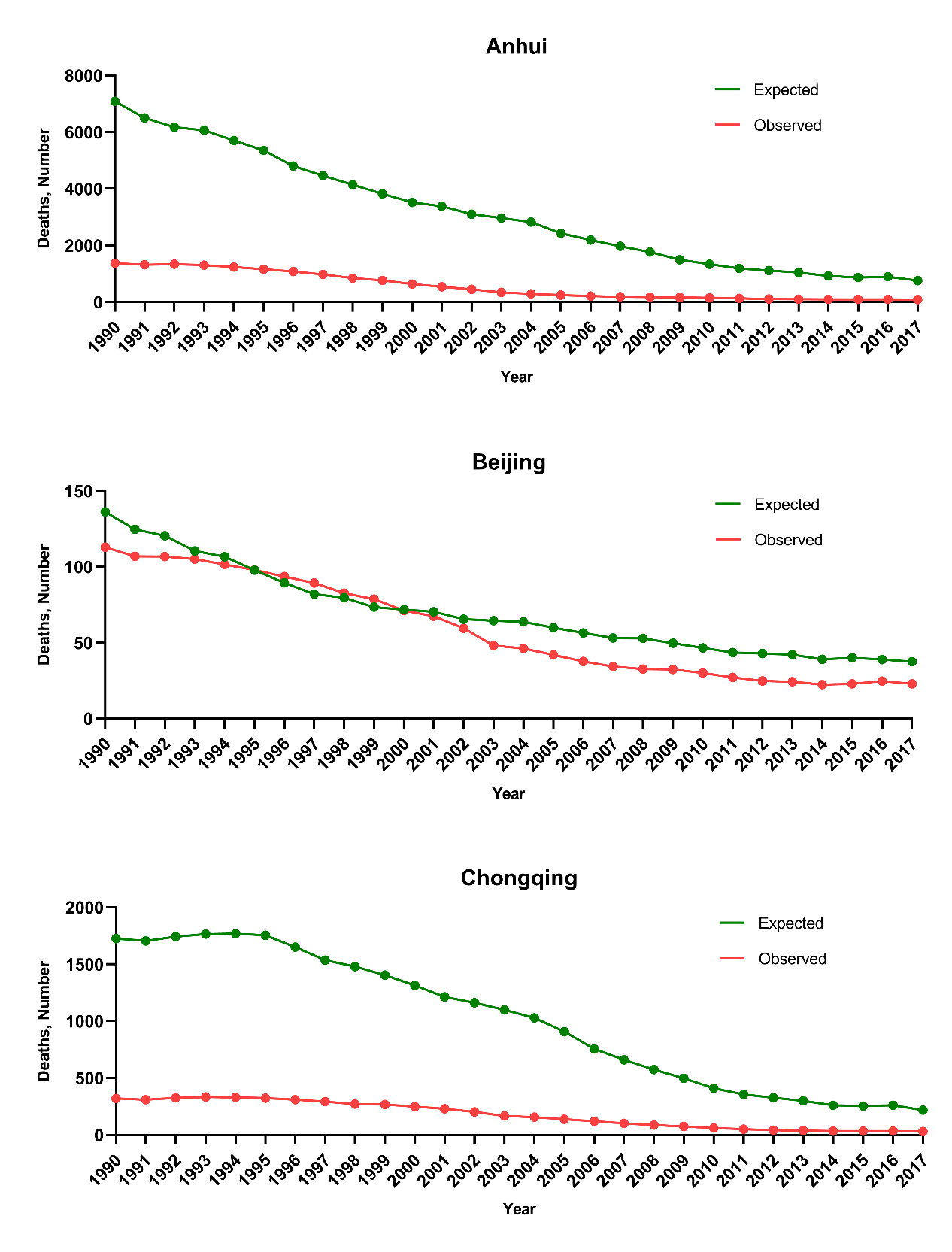


**
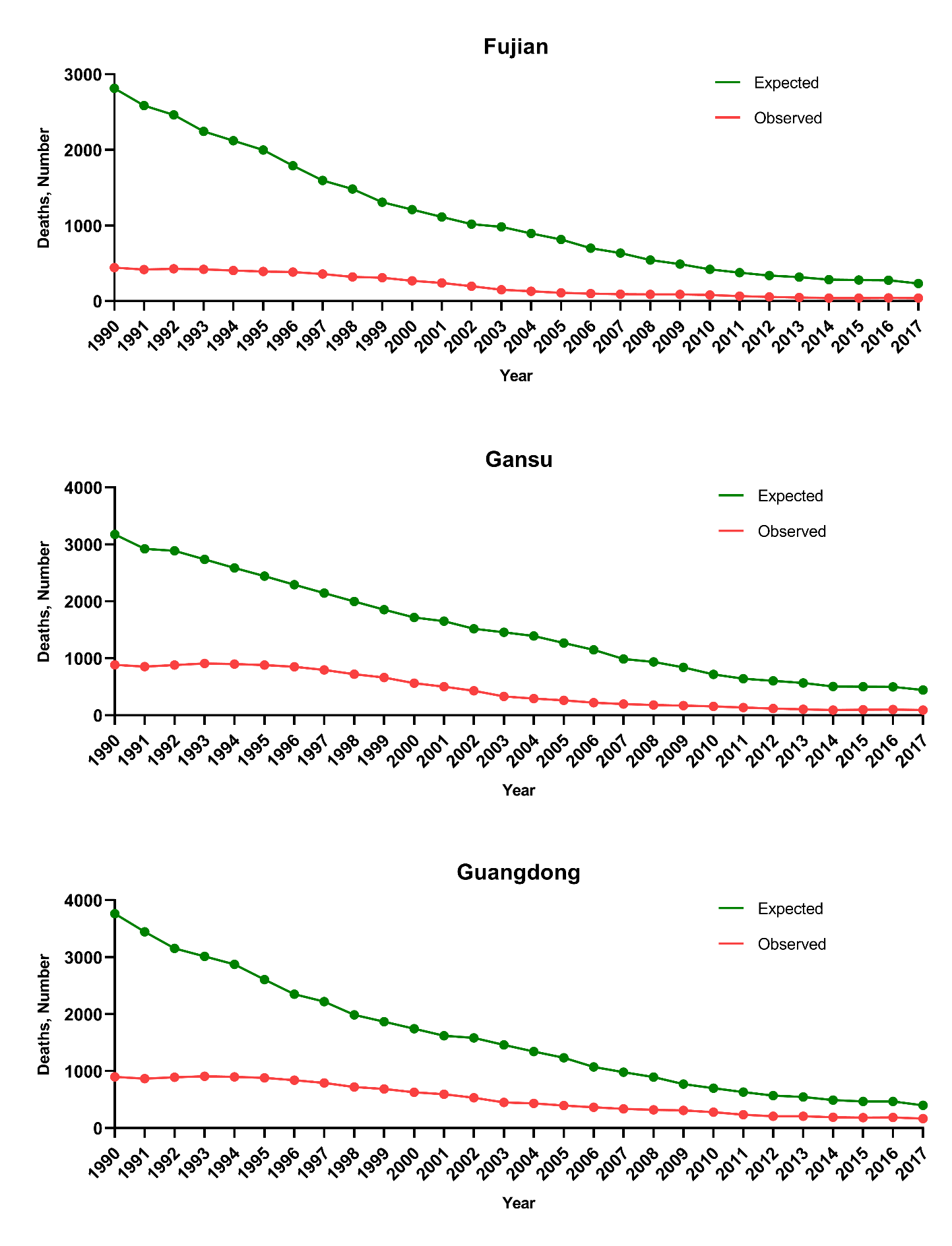
**

**
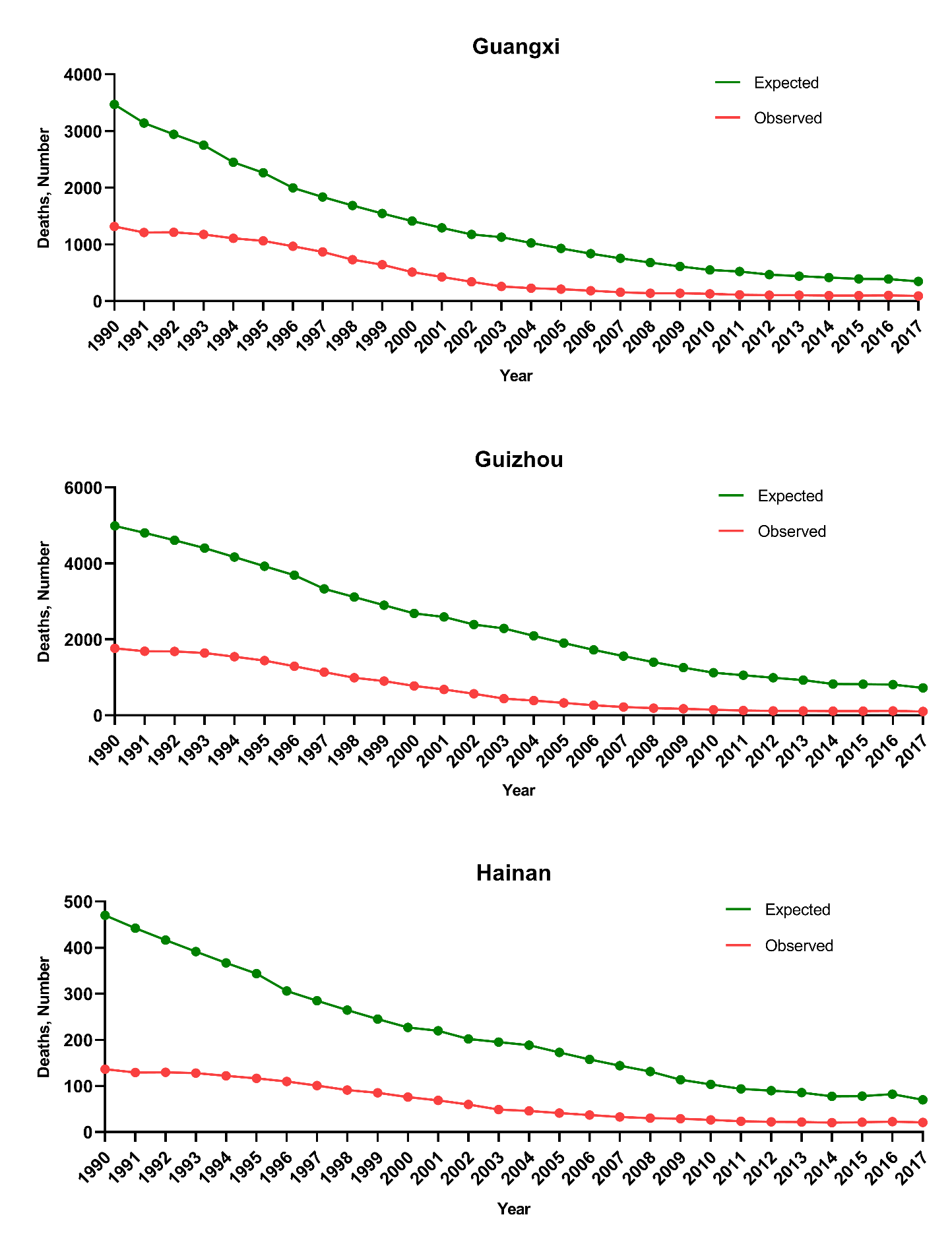
**

**
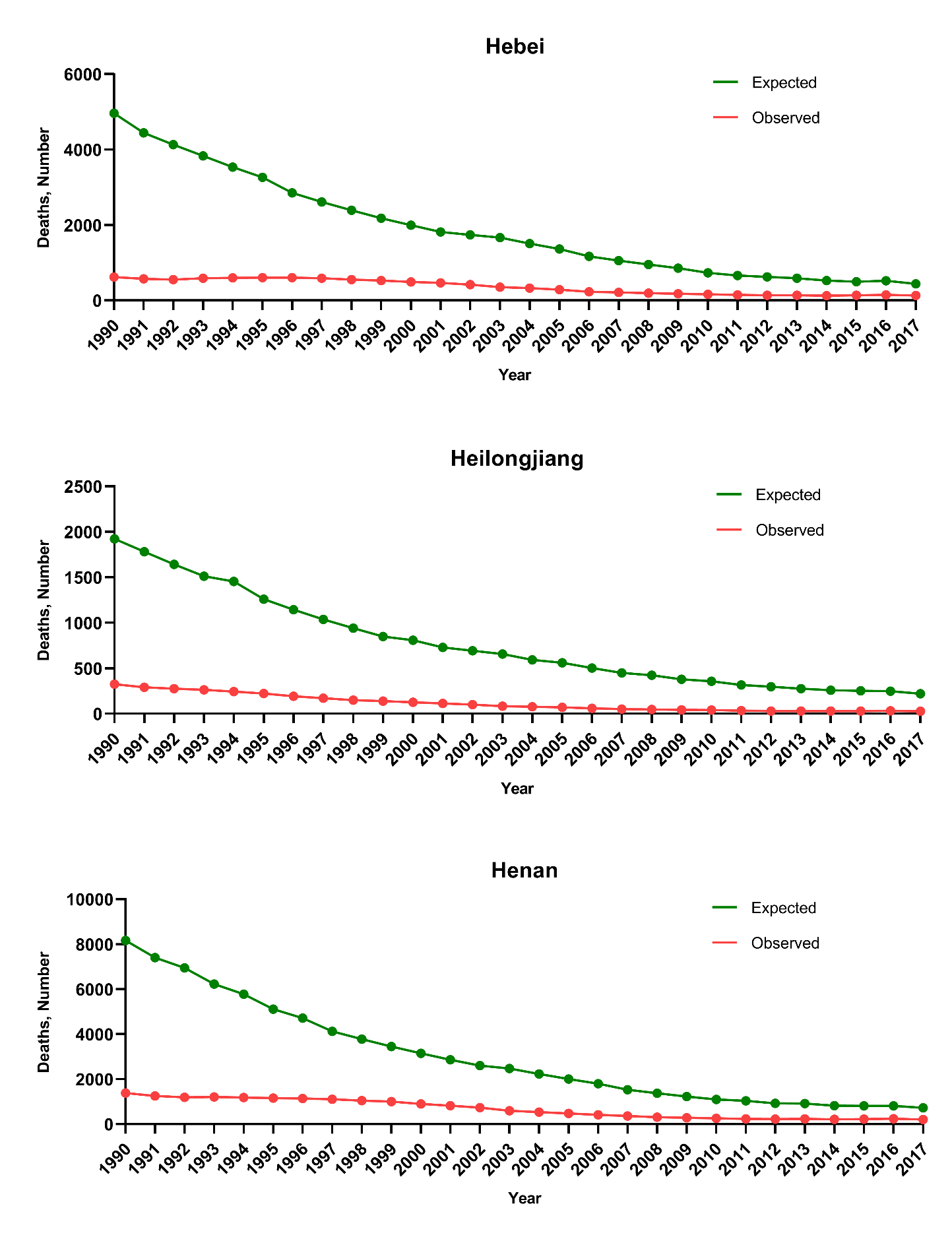
**

**
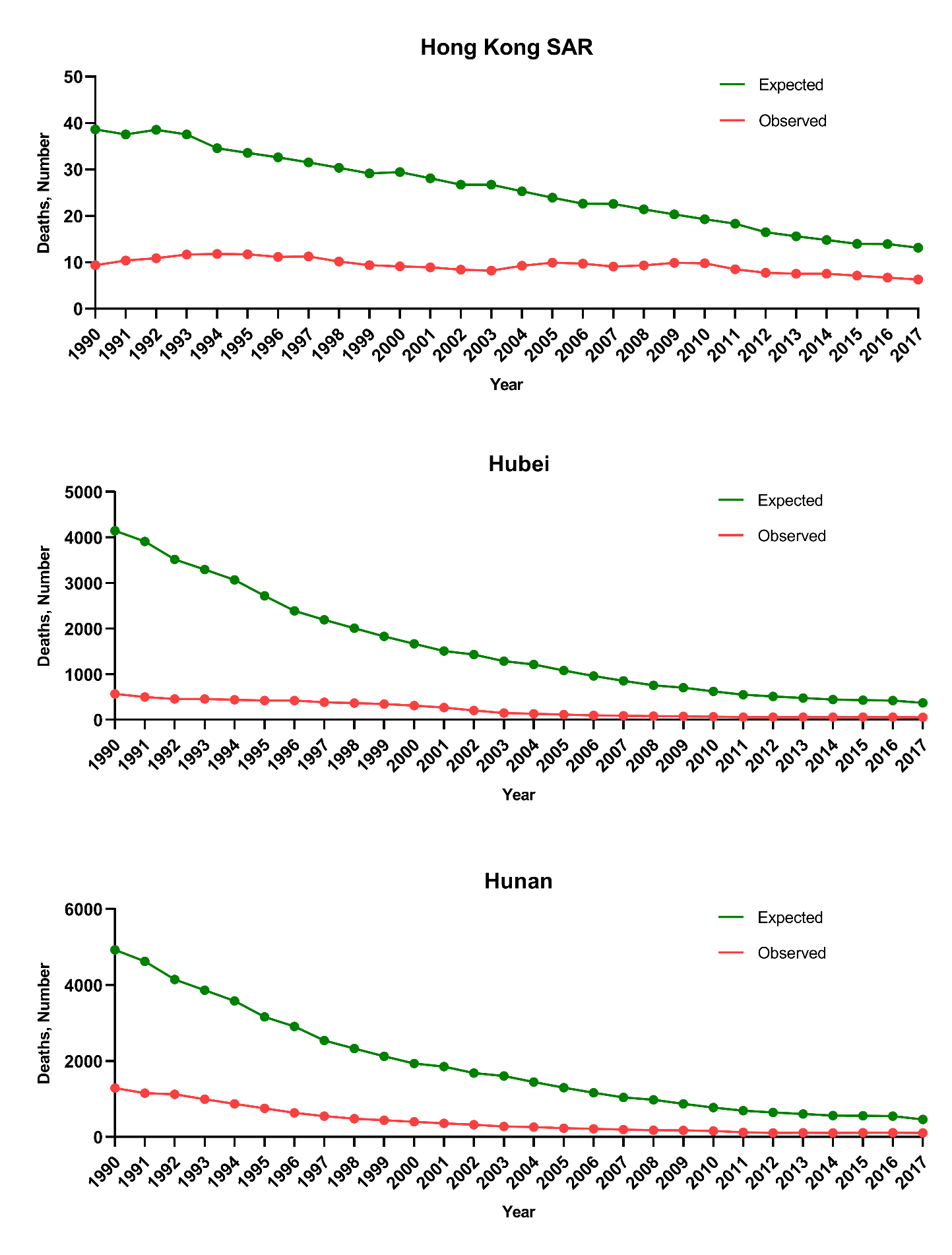
**

**
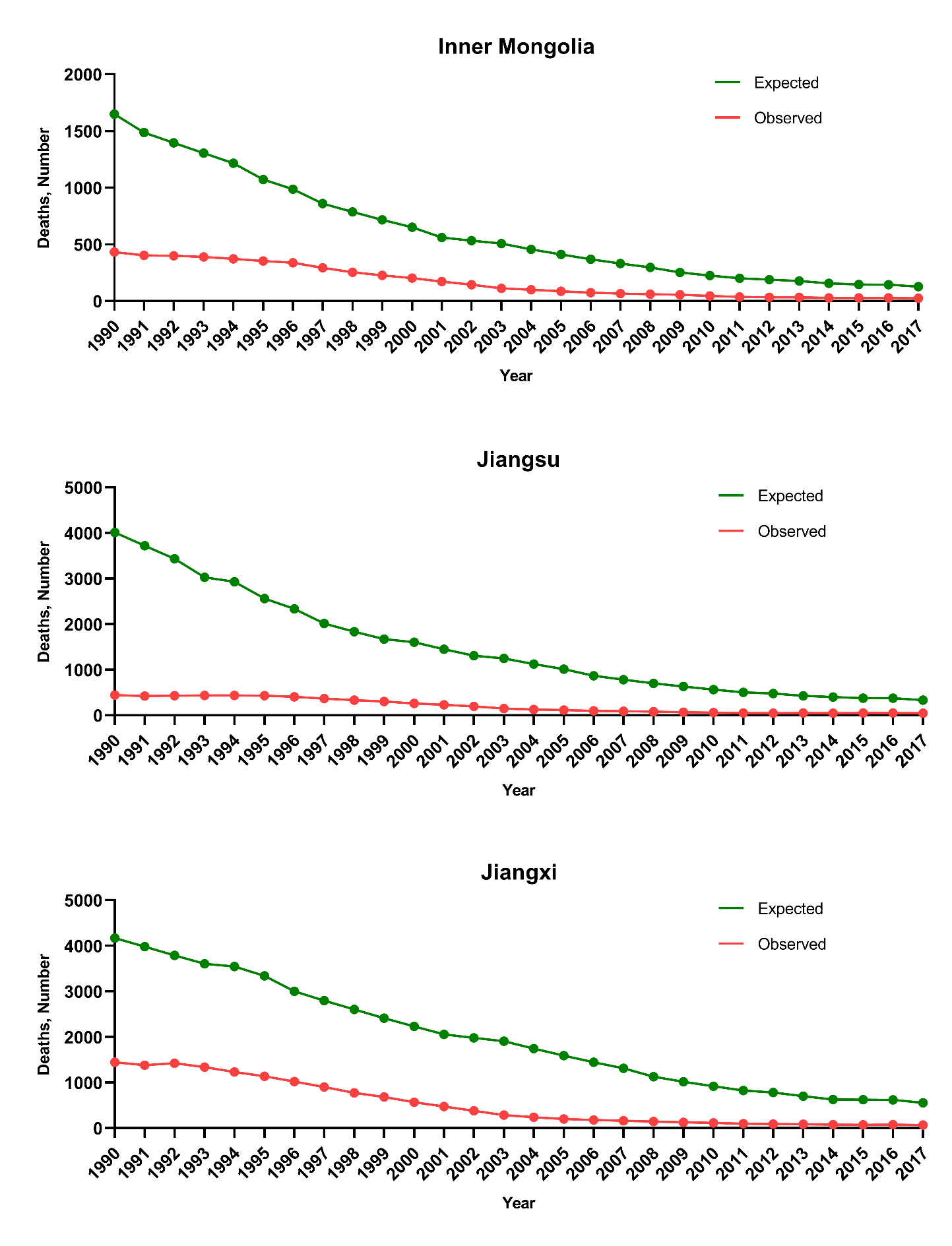
**

**
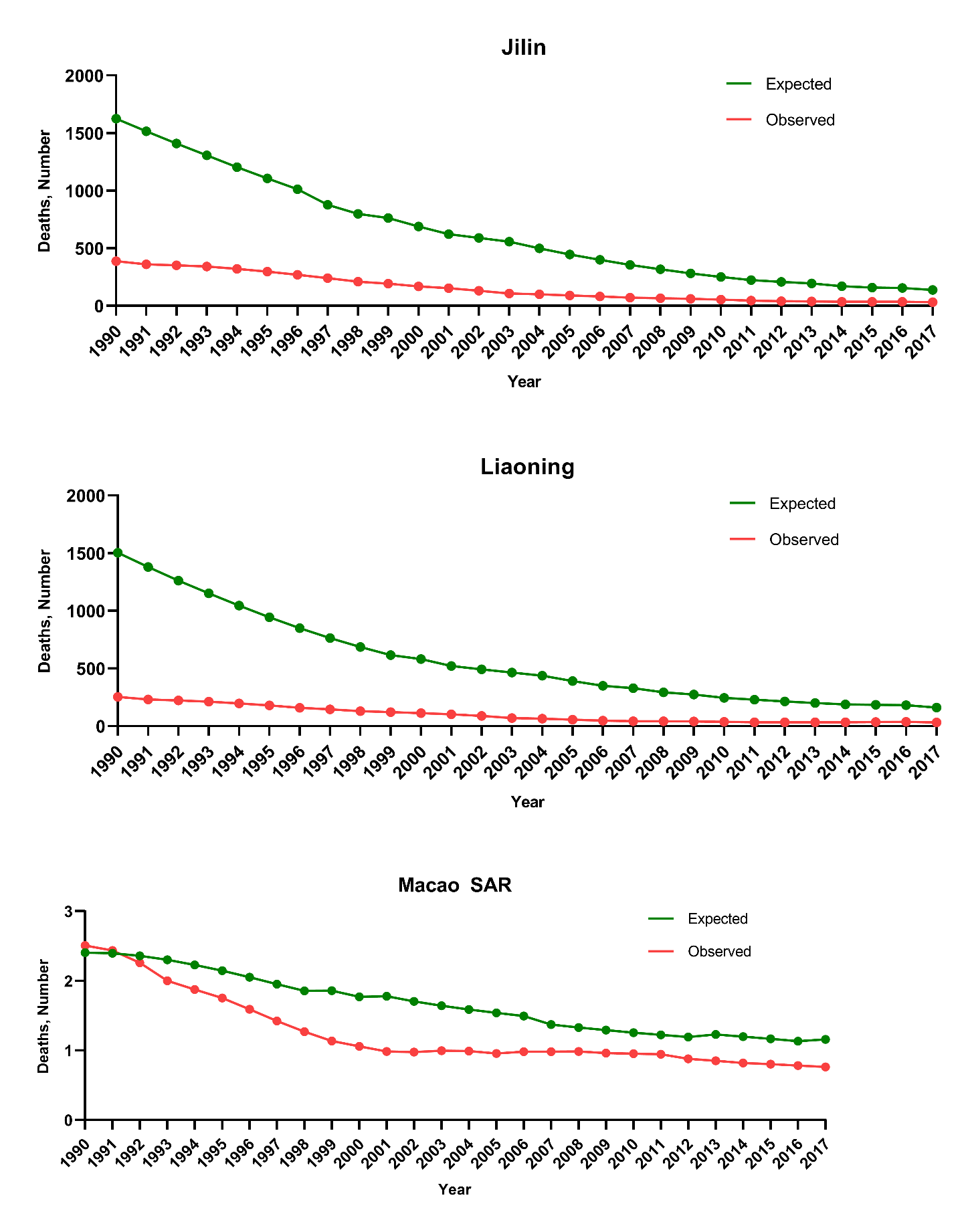
**

**
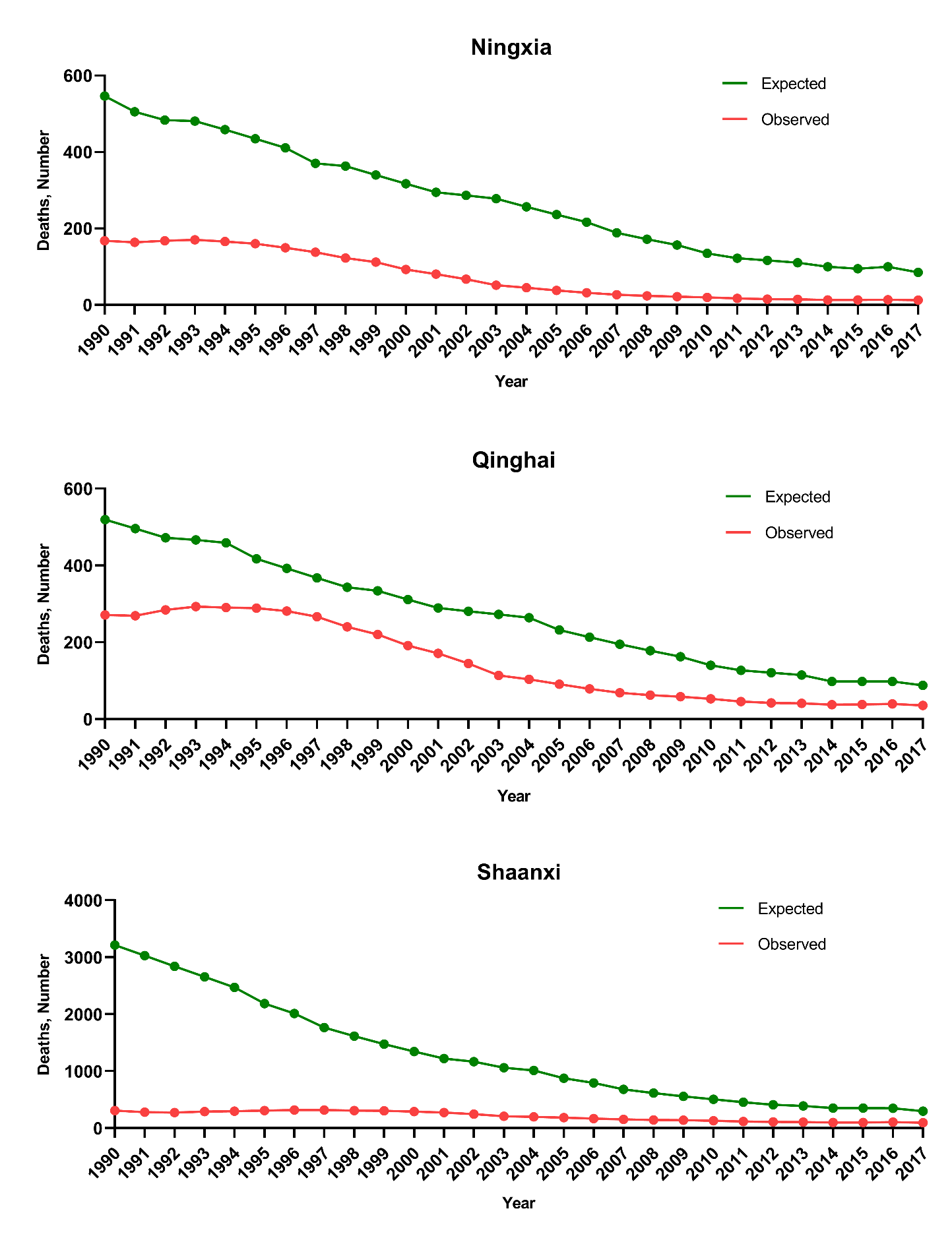
**

**
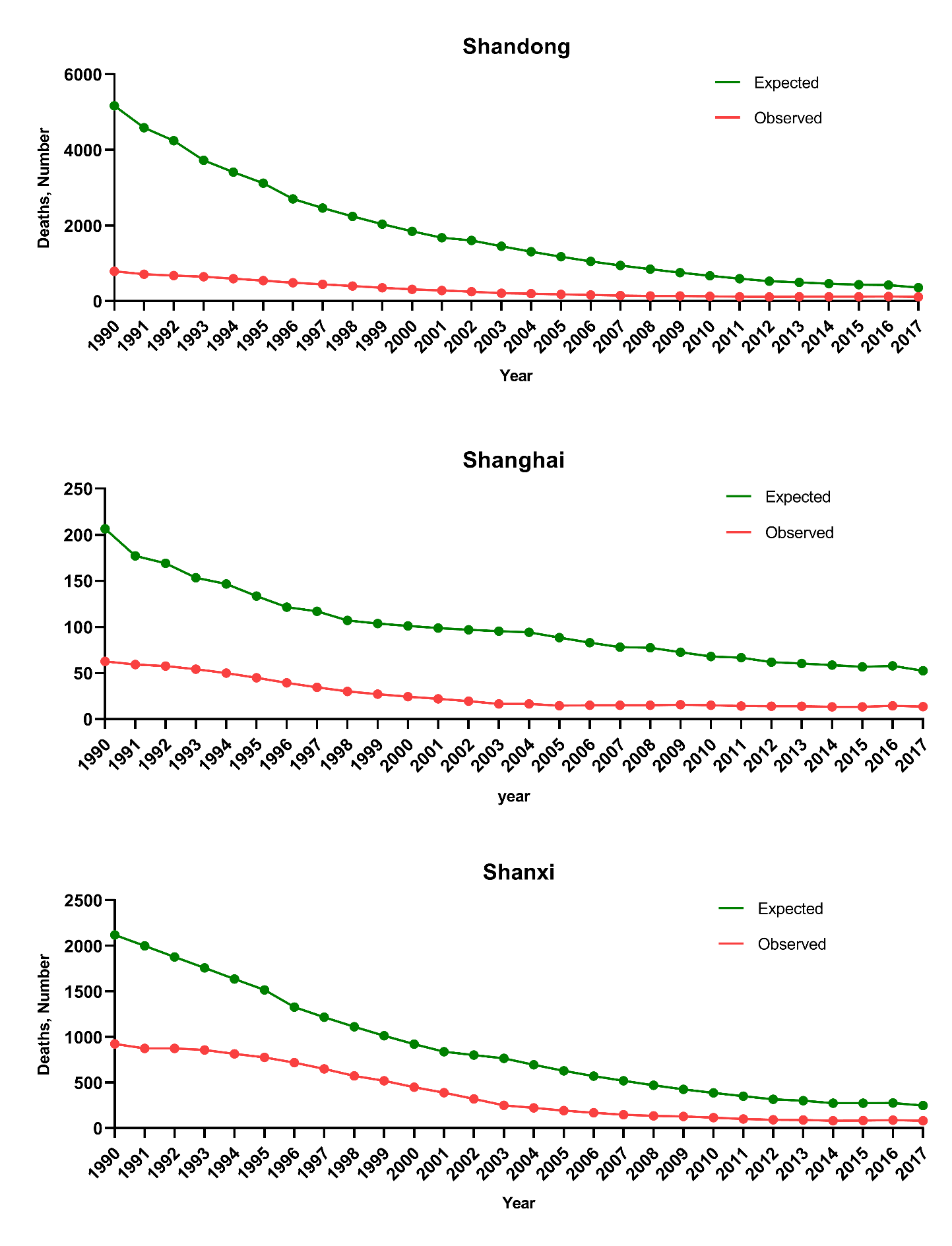
**

**
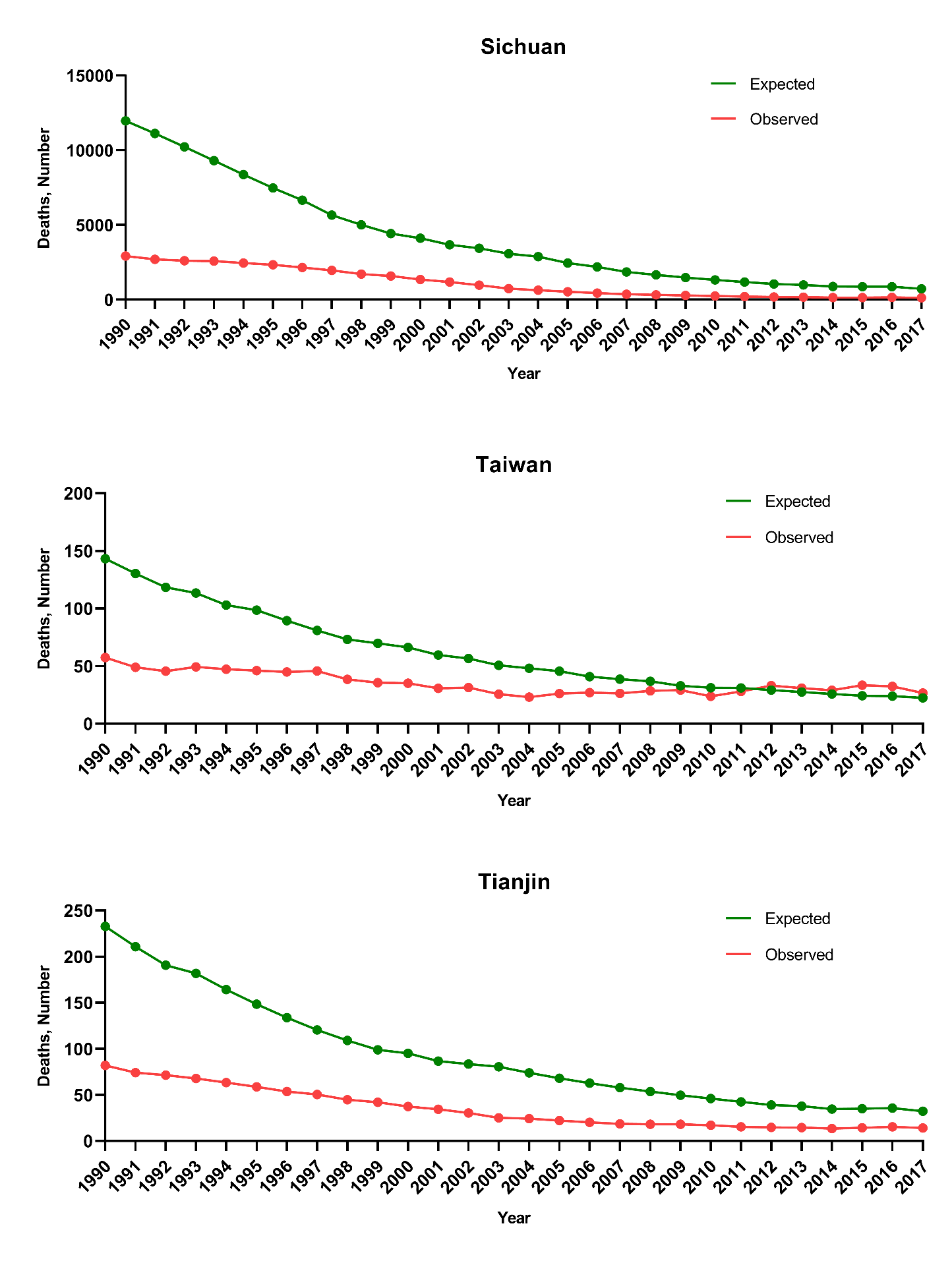
**

**
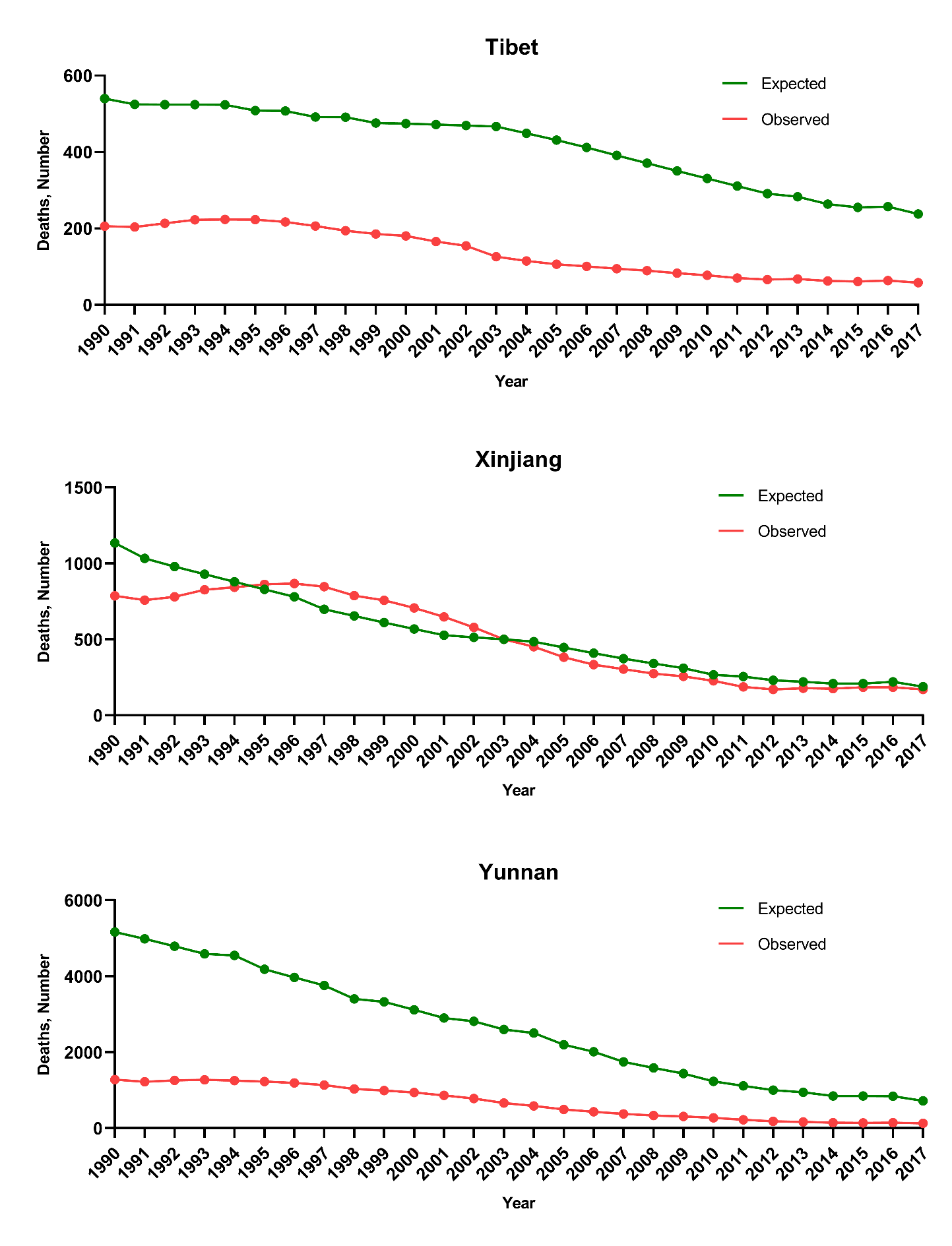
**

**
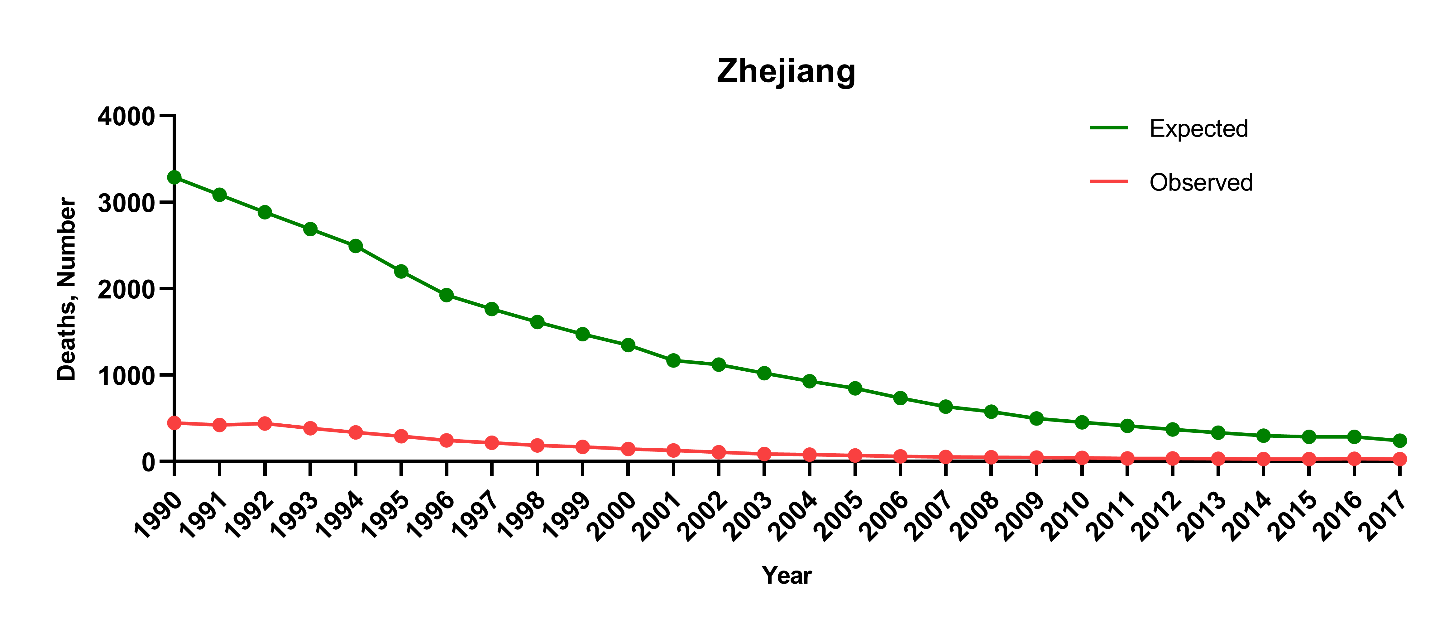
**

**Fig. S2** Cause and number of maternal deaths from 1990 to 2017 in China


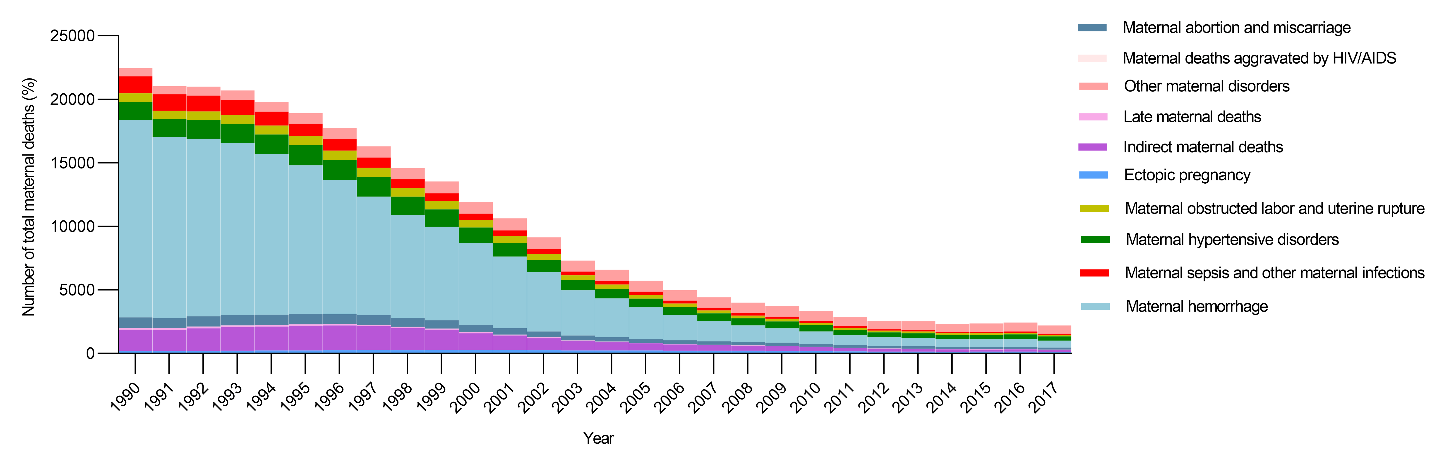


**Fig. S3** Cause and percentage of maternal deaths from 1990 to 2017 in China


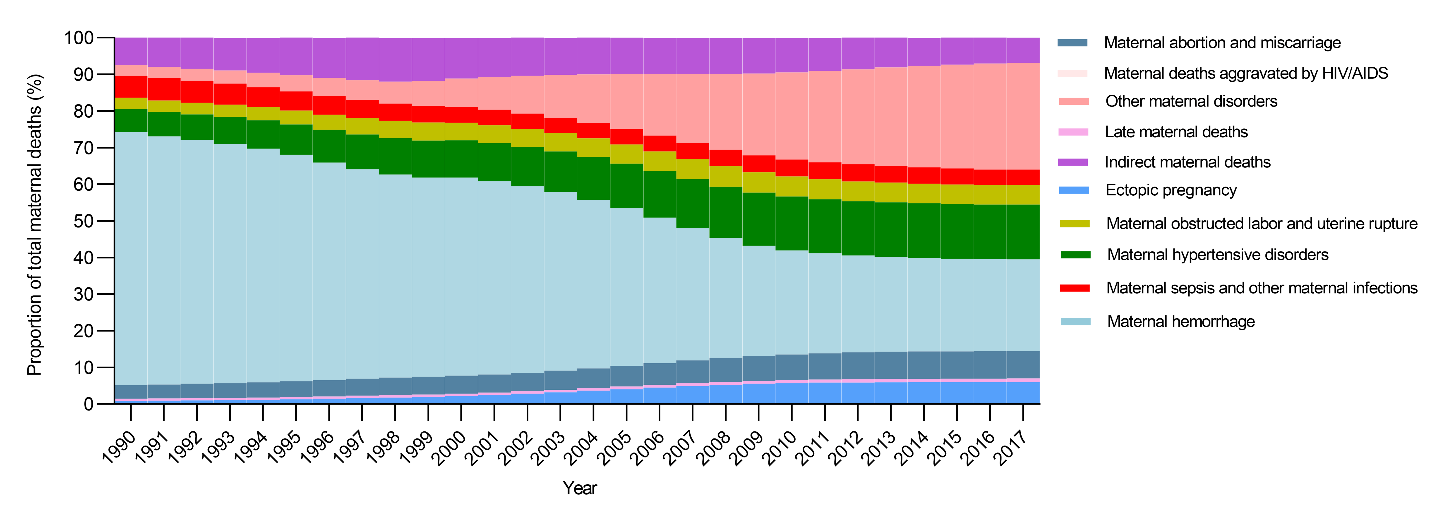

Supplement: Supplementary file 1 — Additional file 1: Table S1. SDI values and quintile groupings for China, 2017. Table S2. Provincial number of maternal deaths by cause in 1990. Table S3. Provincial number of maternal deaths by cause in 2017. Table S4. Provincial number of maternal deaths by cause in each age group in 2017. Figure S1. Observed maternal deaths, and expected maternal deaths probabilistically predicted based on the Socio-demographic Index for the 34 provinces, from 1990 to 2017. Figure S2. Cause and number of maternal deaths from 1990 to 2017 in China. Figure S3. Cause and percentage of maternal deaths from 1990 to 2017 in China. [file 12889_2022_13770_MOESM1_ESM.docx]
